# Pharmacological Inhibition of Lysine-Specific Demethylase 1A Reduces Atherosclerotic Lesion Formation in Apolipoprotein E-Deficient Mice by a Mechanism Involving Decreased Oxidative Stress and Inflammation; Potential Implications in Human Atherosclerosis

**DOI:** 10.3390/antiox11122382

**Published:** 2022-12-01

**Authors:** Simona-Adriana Manea, Mihaela-Loredana Vlad, Alexandra-Gela Lazar, Horia Muresian, Maya Simionescu, Adrian Manea

**Affiliations:** 1 Institute of Cellular Biology and Pathology “Nicolae Simionescu” of the Romanian Academy, 050568 Bucharest, Romania; 2 Cardiovascular Surgery Department, University Hospital Bucharest, 050098 Bucharest, Romania

**Keywords:** NADPH oxidase, oxidative stress, LSD1, histone methylation, atherosclerosis

## Abstract

Dysregulated epigenetic mechanisms promote transcriptomic and phenotypic alterations in cardiovascular diseases. The role of histone methylation-related pathways in atherosclerosis is largely unknown. We hypothesize that lysine-specific demethylase 1A (LSD1/KDM1A) regulates key molecular effectors and pathways linked to atherosclerotic plaque formation. Human non-atherosclerotic and atherosclerotic tissue specimens, ApoE-/- mice, and in vitro polarized macrophages (Mac) were examined. Male ApoE-/- mice fed a normal/atherogenic diet were randomized to receive GSK2879552, a highly specific LSD1 inhibitor, or its vehicle, for 4 weeks. The mRNA and protein expression levels of LSD1/KDM1A were significantly elevated in atherosclerotic human carotid arteries, atherosclerotic aortas of ApoE-/- mice, and M1-Mac. Treatment of ApoE-/- mice with GSK2879552 significantly reduced the extent of atherosclerotic lesions and the aortic expression of NADPH oxidase subunits (Nox1/2/4, p22phox) and 4-hydroxynonenal-protein adducts. Concomitantly, the markers of immune cell infiltration and vascular inflammation were significantly decreased. LSD1 blockade down-regulated the expression of genes associated with Mac pro-inflammatory phenotype. Nox subunit transcript levels were significantly elevated in HEK293 reporter cells overexpressing LSD1. In experimental atherosclerosis, LSD1 mediates the up-regulation of molecular effectors connected to oxidative stress and inflammation. Together, these data indicate that LSD1-pharmacological interventions are novel targets for supportive therapeutic strategies in atherosclerosis.

## 1. Introduction

Compelling clinical and experimental evidence revealed that in a large number of human pathologies, including atherosclerosis-related cardiovascular diseases (CVDs), highly complex networking and functional interactions between genetic variants and epigenetic mechanisms transducing inherited and environmental signals promote transcriptomic and phenotypic alterations [1,2,3]. Contrasting the non-modifiable nature of the innate or acquired genetic information encoded in the DNA sequence, the plasticity of epigenetic changes provides for the potential therapeutic benefit of resetting the dysregulated expression of disease-specific genes employing selective pharmacological interventions [4]. Histone methylation-related epigenetic pathways have emerged as important drug targets in various human malignancies [5]. Hitherto, the potential role of histone methylation-based regulatory mechanisms in the pathoetiology of atherosclerosis has been poorly understood [6,7,8]. Unlike the highly dynamic histone acetylation-induced chromatin relaxation via electric charge neutralization of the lysine residues, histone methylation does not modify the DNA–histone electrostatic interactions. Consequently, different combinations of mono-/di-/tri-methylated histones or modules of histone modifications function as “epi-mutations” that control the recruitment of chromatin-associated proteins, typically referred to as “readers”, which induce or repress the formation of active transcriptional complexes. Two major and highly specialized enzymatic systems known as “writers” (lysine methyltransferases/KMTs, arginine methyltransferases/PRMTs) and “erasers” (lysine demethylases/KDMs) fine-tune the specific signature and the level of histone methylation. Noteworthily, within the epigenetic landscape, H3K4me2, H3K4me3, and H3K79me3 histone marks are typically associated with transcriptional activity, whereas H3K9me2, H3K9me3, H3K27me2, H3K27me3, and H4K20me3 are characteristically related with transcriptional repression of the target genes [5,9].

Lysine-specific demethylase 1 (LSD1/KDM1A), the first identified epigenetic enzyme endowed with histone demethylase activity, is a FAD-dependent amine oxidase that specifically interacts and demethylates mono-/demethylated lysine 4/9 residues on histone H3 (i.e., H3K4me1/2, H3K9me1/2) [10]. As a general principal, the recruitment of LSD1 to gene promoter and enhancer regions triggers transcriptional repression when demethylating H3K4me1/2 (histone marks of active gene expression), also functioning as a key epigenetic driver of gene transcription when acting on H3K9me1/2 (repressive histone marks). Besides histones, LSD1 also regulates the activity of non-histone proteins, including transcription factors, to modulate the gene expression and controls specific protein stability/degradation pathways [11,12]. In addition, LSD1 itself is subjected to post-translational modifications (e.g., methylation, phosphorylation) that significantly impact the biological functions of the enzyme [13].

Alterations in histone methylation patterns have been increasingly associated with the pathology of CVD, both clinically and experimentally [14,15,16]. Hitherto, the functional implication of LSD1 in the regulation of key pathobiological pathways mechanistically linked to atheroma formation, such as oxidative stress and inflammation, has been scantily elucidated. Furthermore, the involvement of LSD1 in mediating the expression of NADPH oxidase (Nox), a master regulator of oxidative stress and inflammation in atherosclerosis, is largely unknown [17,18].

Based on the current knowledge in the field, we hypothesized that LSD1 could play a role in atherogenesis by acting as a key epigenetic regulator of atherosclerosis-related pathological processes, namely Nox up-regulation, oxidative alteration of macromolecules, and inflammation via inflammatory markers. To test this hypothesis, human non-atherosclerotic and atherosclerotic arterial samples, apolipoprotein E-deficient (ApoE-/-) mice, and cultured mouse monocyte (Mon)-derived macrophages (Mac) were investigated.

We provide evidence that archetypal KDM subtypes, including LSD1, are up-regulated in advanced human atherosclerotic lesions, aorta of atherosclerotic ApoE-/- mice, and cultured mouse pro-inflammatory macrophages (M1). In addition, we demonstrate that inhibition of LSD1 (via GSK2879552) (1) significantly reduces atherosclerotic lesion formation, the aortic expression of Nox subtypes, and 4-hydroxynonenal (4-HNE)-protein adduct formation; (2) decreases the markers of immune cell infiltration, inflammation, and vascular remodeling; and (3) significantly down-regulates the expression of selected oxidative stress and pro-inflammatory genes in cultured M1 macrophages.

These results point to LSD1 as a potential pharmacological target in atherosclerosis and indicate that LSD1-oriented treatment could become an important supportive therapeutic option in CVD.

## 2. Materials and Methods

### 2.1. Materials

Unless specifically indicated, general chemicals, reagents, kits, and laboratory disposables were obtained from Sigma-Aldrich, Darmstadt, Germany; Thermo Fisher Scientific/Invitrogen, Waltham, MA, USA; Carl Roth, Karlsruhe, Germany; R&D Systems, Minneapolis, MN, USA; Roche, Basel, Switzerland; Bio-Rad, Herklis, CA, USA; Eppendorf, Hamburg, Germany; and TPP. The tranylcypromine derivative LSD1 pharmacological inhibitor, GSK2879552 (4-[[4-[[[(1R,2S)-2-phenylcyclopropyl]amino]methyl]-1-piperidinyl]methyl]-benzoic acid, C_23_H_28_N_2_O, purity ≥ 98%, CAS No. 1401966-69-5), was purchased from Cayman Chemical, Ann Arbor, MI, USA. Primary and secondary antibodies were from Santa Cruz Biotechnology, Dallas, TX, USA; Abcam, Cambridge, UK; Thermo Fisher Scientific, Waltham, MA, USA; and R&D Systems, Minneapolis, MN, USA. The pCMV6-Entry and pCMV6-KDM1A/LSD1 plasmids were purchased from Origene, Rockville, MD, USA.

### 2.2. Harvesting of Human Non-Atherosclerotic and Atherosclerotic Arterial Tissues

Non-atherosclerotic tissue fragments of ≈ 1–2 mm length derived from the superior thyroid arteries (STA), located in the proximity of the endarterectomized area, and carotid artery-derived atherosclerotic plaques were obtained as discarded biological materials from patients subjected to extended carotid endarterectomy (at University Hospital, Bucharest). Comprehensive Doppler ultrasound imaging and angio-CT interrogation were employed to ascertain the severity of carotid stenosis (i.e., carotid stenosis ≥ 70%) in each patient prior to the surgical procedure. The clinical characteristics of the patients are presented in Table S1 (Supplementary Materials File). The study was performed in accordance with the ethical directives for medical research involving human subjects (The Code of Ethics of the World Medical Association, Declaration of Helsinki). Written informed consent was obtained from all patients enrolled in the study. The experimental protocols were approved by the ethical committee of the Institute of Cellular Biology and Pathology (ICBP) “Nicolae Simionescu” (#11/29.06.2016, #03/07.04.2021).

### 2.3. Set-Up of Experimental Atherosclerosis Mouse Model and Treatment Strategy

Male ApoE-/- mice (B6.129P2-Apoe^tm1Unc^/J; strain number 002052) and C57BL/6J mice (strain number 000664) obtained from The Jackson Laboratory were employed. The mice were exposed to 12 h of light/dark cycles and had access to standard rodent diet and water ad libitum. At 8 weeks of age, ApoE-/- mice were divided into two groups: (1) receiving a normal diet (ND, control, *n* = 15) and (2) fed a high-fat, cholesterol-rich diet (HD, *n* = 30) for 10 weeks to accelerate the development of atherosclerotic lesions throughout the aorta as previously described [19]. After 10 weeks on a normal or atherogenic diet, the mice were further distributed into three experimental groups (*n* = 15/group) to receive via intraperitoneal (i.p.) injection 5 mg/kg GSK2879552 pharmacological inhibitor of LSD1 or its vehicle (5% DMSO + 95% PBS, pH 7.4), for 4 weeks. The three groups were (1) ApoE-/- (ND) + vehicle, (2) ApoE-/- (HD) + vehicle, and (3) ApoE-/- (HD) + GSK2879552.

The dose and the procedure of GSK2879552 treatment of mice were established in accordance with the data derived from preceding reports [20,21]. The animal studies were conducted in agreement with the guidelines of EU Directive 2010/63/EU, and the associated experimental protocols were approved by the ethical committee of the ICBP “Nicolae Simionescu” (#04/07.04.2021).

### 2.4. Cell Culture Experimental Design

Cultured resting (M0) and polarized pro-inflammatory (M1) and anti-inflammatory (M2) mouse Mon-derived Mac were employed to examine the expression pattern of archetypal KDM subtypes and the potential implication of LSD1 in mediating the up-regulation of oxidative stress- and inflammation-related genes. Mon were freshly isolated by negative selection procedure from the spleen of C57BL/6J mice (*n* = 40) employing an EasySep mouse monocyte isolation kit (Stemcell Technologies, Vancouver, BC, Canada). Mon-to-Mac differentiation (M0) and polarization were performed as previously described [22] employing 100 ng/mL LPS + 20 ng/mL IFNγ to induce the M1-like Mac or 20 ng/mL IL-4 to promote the M2-like Mac phenotype. The cells were further exposed for 24 h to medium alone (M0–Mac) or medium with polarization factors (e.g., LPS + IFNγ/M1-Mac; IL-4/M2-Mac) in the absence or presence of 5μM LSD1-inhibitor (GSK2879552).

A human embryonic kidney 293 (HEK293) cell line obtained from the American Type Culture Collection (ATCC), generally acknowledged for its high transfection efficiency, was employed in transfection assays.

### 2.5. Histology and Microscopic Examination

The overall structural alterations and the cell identification were assessed by hematoxylin–eosin (H&E) staining of human non-atherosclerotic STA and carotid artery-derived atherosclerotic tissue samples. After surgical harvesting, the arterial specimens were rinsed in PBS (pH 7.4), fixed in 4% buffered-paraformaldehyde solution, cryoprotected, and embedded in optimal cutting temperature compound. Cryosections (5 μm thick) of STA and carotid artery were mounted onto SuperFrost Plus microscope slides (Thermo Scientific, Waltham, MA, USA), stained with H&E solution according to manufacturer’s protocol (Carl Roth, Karlsruhe, Germany), and examined and photographed with a Zeiss Axio Observer microscope (Carl Zeiss, Jena, Germany).

### 2.6. Assessment of Plasma Total Cholesterol and Triglyceride Levels in Mice

At sacrifice, blood samples (≈1 mL) were collected from mice via cardiac puncture in EDTA-coated tubes (Becton Dickinson Vacutainer spray-coated EDTA tubes) and subjected to plasma preparation through centrifugation. Total cholesterol and triglyceride levels were determined spectrophotometrically in the plasma of mice using standard colorimetric kits (Dialab, Vienna, Austria).

### 2.7. Assessment of Atherosclerotic Lesion Formation in Mice

The formation of atherosclerotic lesions throughout the aorta of ApoE-/- mice was determined by en face Oil Red O (ORO) staining as previously described [22]. ImageJ software (NIH Image, Bethesda, MD, USA) was employed to quantify the extent of ORO positive staining area.

### 2.8. Real-Time Polymerase Chain Reaction Assay (Real-Time PCR)

Total RNA was purified from human and mouse arterial tissues and cultured Mac/HEK293 cells using column-based RNA isolation kits (Qiagen/tissue, Hilden, Germany, Sigma/cells, Darmstadt, Germany). Prior to RNA purification, human and mouse tissues were washed with ice-cold PBS (pH 7.4), resuspended in QIAzol lysis reagent, and subjected to glass bead homogenization (BioSpec). MMLV reverse-transcriptase was employed to synthesize the complementary DNA strand (cDNA) according to the manufacturer’s protocol (Thermo Fisher Scientific, Waltham, MA, USA). The amplification of cDNA was assessed by real-time PCR assay employing the SYBR Green I fluorescent probe (LightCycler 480 II thermocycler, Roche, Basel, Switzerland). The comparative C_T_ method [23] was used to quantify the mRNA expression using the β-actin mRNA level for internal normalization. The sequences of the oligonucleotide primers used to analyze the mRNA expression levels of Nox components and inflammatory mediators are included in Table S2 (Supplementary Materials File). The oligonucleotide primer sequences used to analyze the expression of KDM transcripts were derived from Origene, Rockville, MD, USA.

### 2.9. Western Blot Assay

The total protein extracts derived from human and mouse arterial tissues and the cultured HEK293 cells were prepared as previously described [22]. After surgical harvesting, the tissue specimens were rinsed in ice-cold PBS (pH 7.4), resuspended in RIPA buffer containing a protease inhibitor cocktail (Sigma, Darmstadt, Germany), and subjected to mechanical homogenization (BioSpec, glass bead diameter: 1 mm). Cultured cells were washed with PBS (pH 7.4) and collected in RIPA buffer containing protease inhibitors. After dilution in 2 × Laemmli’s buffer (Serva, Odessa, TX, USA), the tissue and cell lysates were incubated for 20 min at 95 °C, run on SDS-PAGE (30 µg protein/lane), and transferred onto nitrocellulose membranes (Bio-Rad, Herklis, CA, USA). The primary antibodies were as follows: LSD1/KDM1A (rabbit monoclonal, ab129195, dilution 1:1000), Nox1 (rabbit polyclonal, ab131088, dilution 0.5 μg/mL), Nox2 (mouse monoclonal, sc-130543, dilution 1:200), Nox4 (rabbit polyclonal, sc-30141, dilution 1:200), p22phox (mouse monoclonal, sc-271262, dilution 1:200), 4-hydroxynonenal/4-HNE (mouse monoclonal, MAB3249, dilution 1 µg/mL), CD68 (mouse monoclonal, sc-130543, dilution 1:200), CD80 (mouse monoclonal, sc-376012, dilution 1:200), CD86 (mouse monoclonal, sc-19617, dilution 1:200), TLR2 (mouse monoclonal, sc-21760, dilution 1:200), TLR4 (mouse monoclonal, sc-52962, dilution 1:200), NOS2 (mouse monoclonal, sc-7271, dilution 1:200), VCAM-1 (mouse monoclonal, MA5-11447, dilution 1:500), MMP2 (mouse monoclonal, sc-13594, dilution 1:200), MMP9 (mouse monoclonal, sc-393859, dilution 1:200), and β-actin (mouse monoclonal, sc-47778, dilution 1:500). Anti-rabbit IgG-HRP (sc-2370, dilution 1:5000) and anti-mouse IgG-HRP (sc-2031, dilution 1:5000) were used as secondary antibodies. The chemiluminescence signal of the protein bands was detected with the ImageQuant LAS 4000 system (Fujifilm, Port Area, Tokyo, metropolitan area). The TotalLab software was employed for densitometric analysis using the β-actin protein level for internal normalization.

### 2.10. Transfection Assay

HEK293 cells were seeded at a density of 1 × 10^5^ cells per well into 12-well tissue culture plates 24 h prior to transfection. Transient transfection was performed employing Viromer Red reagent using 11 ng/μL pCMV6-Entry (empty vector, control) or pCMV6-LSD1/KDM1A (NM_015013) mammalian expression vectors (Origene, Rockville, MD, USA). At 24 h post-transfection, the cells were subjected to neomycin selection for 3 days. The neomycin-resistant cells (i.e., cells efficiently transfected with the pCMV6 expression vectors) were used for further experiments.

### 2.11. Statistical Analysis

Data obtained from a minimum of three independent experimental conditions were expressed as mean ± standard deviation. Statistical analysis was performed by *t*-test and one-way analysis of variance (ANOVA) followed by Tukey’s post hoc test; *p* < 0.05 was considered as statistically significant.

## 3. Results

### 3.1. LSD1 Expression Is Up-Regulated in Atherosclerotic Human Carotid Arteries

**Figure 1:**
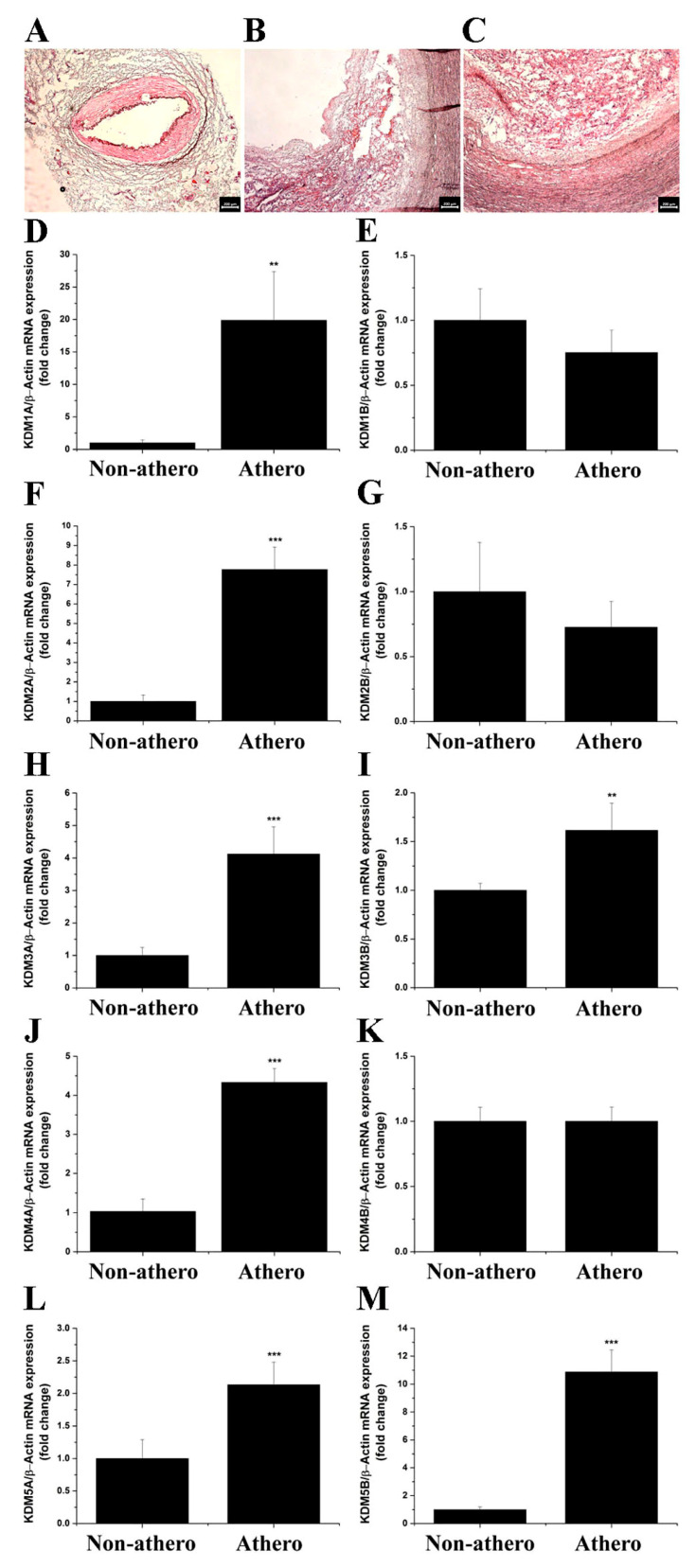
The mRNA expression levels of archetypal KDM subtypes are significantly up-regulated in atherosclerotic human carotid arteries. Representative H&E staining images taken under 5× magnification of (**A**) non-atherosclerotic STA and (**B**,**C**) carotid artery-derived atherosclerotic sections obtained from a study patient undergoing extended carotid endarterectomy. (**D**–**M**) Real-time PCR-based gene expression analysis depicting the augmented mRNA levels of LSD1 (KDM1A), KDM2A, KDM3A, KDM3B, KDM4A, KDM5A, and KDM5B in human atherosclerotic lesions. *n* = 4–9, ** *p* < 0.01, *** *p* < 0.001. *p*-values were taken in relation to non-atherosclerotic condition. Non-athero, non-atherosclerotic specimens; Athero, atherosclerotic specimens.

**Figure 2:**
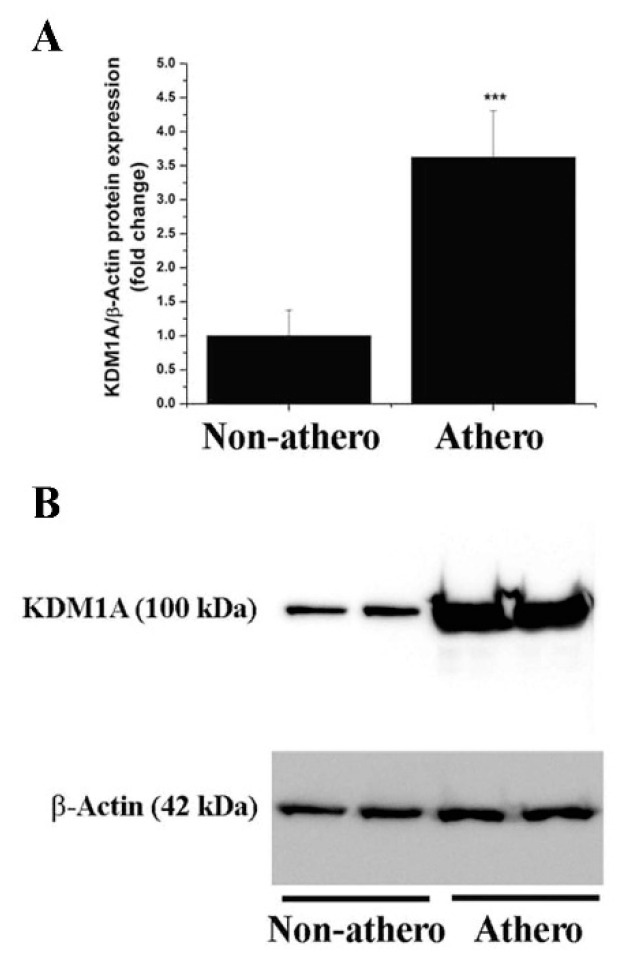
The protein level of LSD1 (KDM1A) is significantly elevated in atherosclerotic human carotid arteries. (**A**) Western blot assay-associated densitometric analysis depicting the LSD1 (KDM1A) relative protein expression level in human non-atherosclerotic STA and carotid artery-derived atherosclerotic tissue specimens. (**B**) Representative immunoblot showing the up-regulation of LSD1 (KDM1A) protein level in atherosclerotic tissue derived from human carotid arteries as compared to non-atherosclerotic arterial samples. *n* = 4–7, *** *p* < 0.001. *p*-value was taken in relation to non-atherosclerotic condition.

First, we examined whether KDM subtype levels are dysregulated in human atherosclerosis, focusing on the expression profiling of a high-priority KDM panel. The latter included LSD1 expression in samples of atherosclerotic tissue derived from human carotid arteries and non-atherosclerotic tissue obtained from STA, as controls. Microscopic examination of the tissue sections (H&E staining) showed typical structural alterations of the fibro-lipid advanced atherosclerotic plaques and the presence of immune cell infiltration. No structural alterations and atherosclerotic lesions were detected in the STA specimens (Figure 1A–C). The initial gene expression profiling assays demonstrated significant increases in the mRNA expression levels of LSD1/KDM1A (≈20-fold), KDM2A (≈8-fold), KDM3A (≈4-fold), KDM3B (≈1.5-fold), KDM4A (≈4-fold), KDM5A (≈2-fold), and KDM5B (≈11-fold) in human atherosclerotic lesions as compared with non-atherosclerotic control levels. No significant changes in KDM1B, KDM2B, and KDM4B transcript levels were detected in atherosclerotic tissues as compared with non-atherosclerotic STA conditions (Figure 1D–M). Moreover, Western blot assays revealed significantly elevated protein levels of LSD1/KDM1A (≈3.5-fold) in tissue homogenates derived from atherosclerotic lesions as compared to controls, the non-atherosclerotic tissue (Figure 2). Collectively, these data demonstrated the association between up-regulated mRNA/protein levels of LSD1/KDM1A and other KDM subtypes in advanced human atherosclerotic lesions. Considering the current status of knowledge in the field, one could predict that LSD1/KDM1A modulates atherosclerosis-relevant signaling pathways.

### 3.2. The Gene and Protein Expression Levels of LSD1 Are Up-Regulated in the Aorta of Atherosclerotic Mice

**Figure 3:**
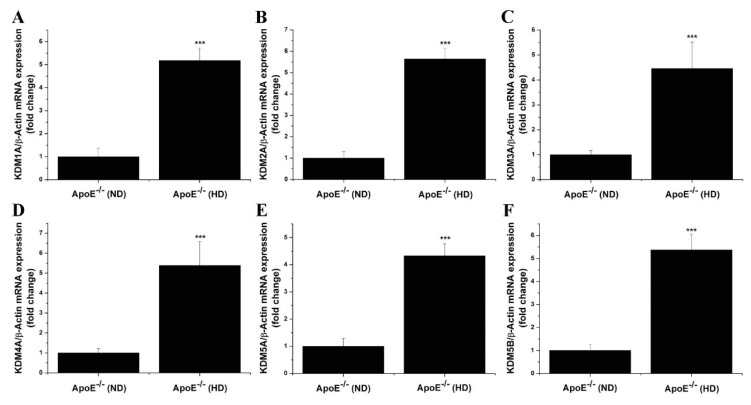
The mRNA levels of LSD1 and selective KDM subtypes induced in an experimental animal model of atherosclerosis. (**A**–**F**) Real-time PCR gene expression profiling analysis indicating the up-regulation of LSD1 (KDM1A), KDM2A, KDM3A, KDM4A, KDM5A, and KDM5B transcript levels in the atherosclerotic aorta of ApoE-/- mice. *n* = 4, *** *p* < 0.001. *p*-value was taken in relation to ApoE-/- (ND) condition.

**Figure 4:**
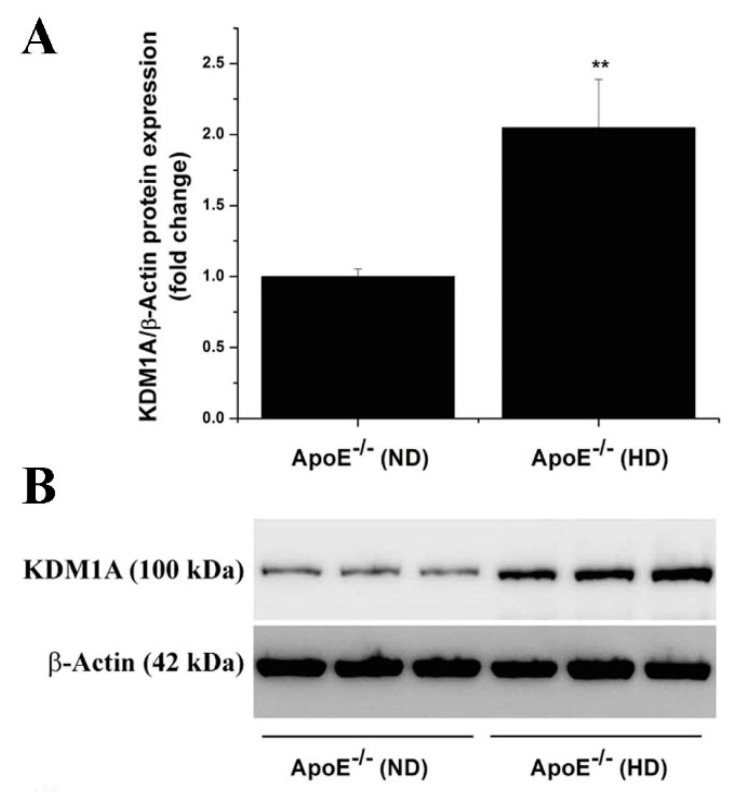
The relative abundance of LSD1 (KDM1A) protein is elevated in atherosclerotic ApoE-/- mice. (**A**) Western blot assay-associated densitometric analysis indicating the augmented level of LSD1 (KDM1A) protein in the atherosclerotic aorta of ApoE-/- mice after 14 weeks on high-fat, cholesterol-rich diet. (**B**) Representative immunoblot showing the elevated level of LSD1 (KDM1A) protein in the aorta of ApoE-/- (HD) as compared with ApoE-/- (ND) mice. *n* = 3, ** *p* < 0.01. *p*-value was taken in relation to ApoE-/- (ND) condition.

Next, we examined the occurrence of a similar KDM subtype expression pattern in the aorta of ApoE-/- mice fed a high-fat, cholesterol-rich diet (HD) for 14 weeks, an experimental set-up partially resembling human atherosclerosis [19]. The gene expression profiling demonstrated significant increases in the mRNA levels of LSD1/KDM1A (≈5-fold), KDM2A (≈5.5-fold), KDM3A (≈4.5-fold), KDM4A (≈5.5-fold), KDM5A (≈4-fold), and KDM5B (≈5-fold) in the aorta of atherosclerotic ApoE-/- (HD) mice compared to the values obtained for ApoE-/- (ND) animals (Figure 3). A significantly elevated LSD1/KDM1A protein level (≈2 -fold) was determined by protein analysis assays in tissue homogenates derived from the atherosclerotic aorta of ApoE-/- (HD) mice as compared to ApoE-/- (ND) animals (Figure 4). These data indicated that various KDM subtypes, including LSD1, could control similar pathological mechanisms in both human and experimental atherosclerosis.

### 3.3. Pharmacological Inhibition of LSD1 Activity by GSK2879552 Reduces Atherosclerotic Lesion Formation in the Aorta of Hypercholesterolemic Mice

**Figure 5:**
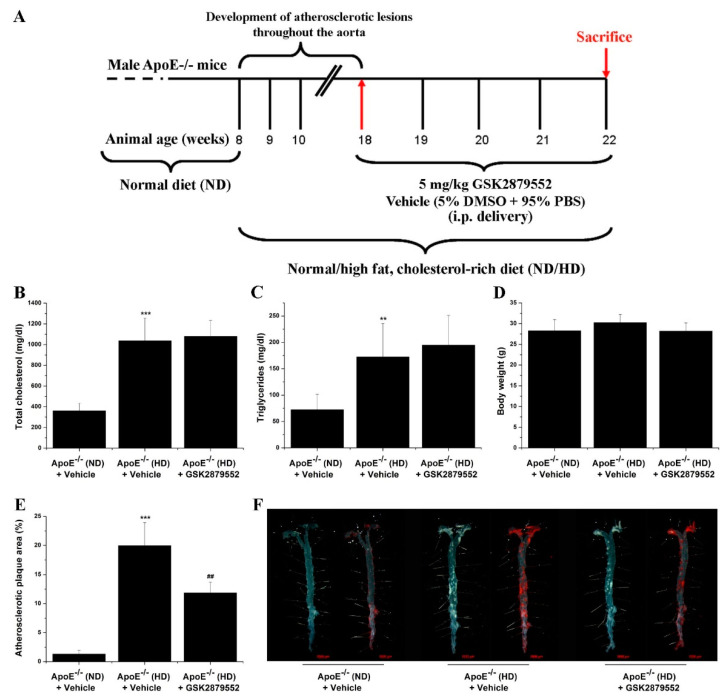
LSD1-related signaling pathways contribute to atherosclerotic lesion formation in ApoE-/- mice. (**A**) Schematic depiction of the general experimental set-up to promote atherosclerotic lesion formation throughout the aorta of ApoE-/- mice and the animal treatment strategy with the GSK2879552 pharmacological inhibitor. (**B**–**D**) Evaluation of plasma total cholesterol and triglyceride levels and body weight of ApoE-/- mice at the end of the treatment procedure. (**E**) Quantification of atherosclerotic lesion area in each animal group. (**F**) Representative images of en face Oil Red O (ORO) staining depicting the extent of atherosclerotic lesion formation throughout the aorta of ApoE-/- mice. The aortas were photographed in the absence and presence of the lipid staining solution. *n* = 6, ** *p* < 0.01, *** *p* < 0.001. *p*-values were taken in relation to ApoE-/- (ND) condition. ## *p* < 0.01. *p*-value was taken in relation to ApoE-/- (HD) condition.

To explore the potential functional implication of LSD1 in the process of atheroma formation in ApoE-/- mice, we employed a clinically approved LSD1 pharmacological inhibitor, GSK2879552. After 10 weeks on a normal or high-fat, cholesterol-rich diet, male ApoE-/- mice were randomly distributed into three experimental groups to receive GSK2879552 or its vehicle for 4 weeks. A schematic depiction of the experimental design and treatment strategy is presented in Figure 5A. As expected, significant increases in total plasma cholesterol (≈3-fold) and triglyceride (≈2.5-fold) levels were determined in the HD mice after 14 weeks of an atherogenic diet compared to control mice fed a normal diet. No significant changes in these plasma parameters and body weight were detected following long-term pharmacological blockade of LSD1 by GSK2879552 in HD mice compared to vehicle-treated mice (Figure 5B–D). As revealed by Oil Red O staining, the extent of the atherosclerotic lesional area was significantly augmented (≈15-fold) throughout the aorta of ApoE-/- (HD) mice as compared to ApoE-/- (ND) control mice. Long-term administration of GSK2879552 significantly decreased (≈40%) the progression of atherosclerotic lesions in ApoE-/- (HD) mice as compared with vehicle-treated animals (Figure 5E,F). These data suggest that inhibition of up-regulated LSD1 in hypercholesterolemic mice negatively interferes with LSD1-related signaling pathways that are mechanistically linked to atherosclerotic plaque formation, regardless of increased plasma total cholesterol and triglyceride levels.

### 3.4. LSD1-Dependent Signaling Mediates the Up-Regulation of Nox Subunit Expression in the Atherosclerotic Mice Aorta

**Figure 6:**
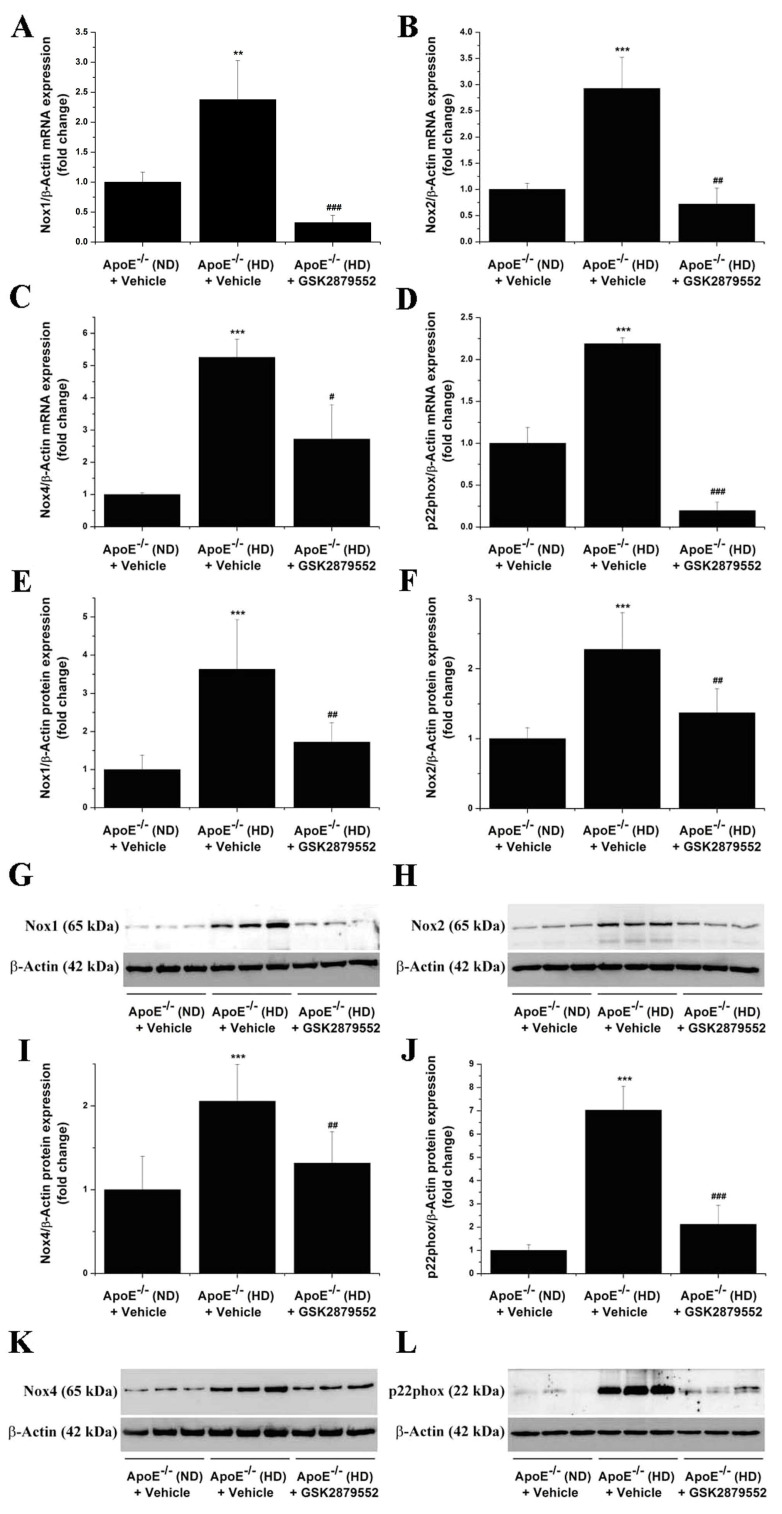
Long-term pharmacological inhibition of LSD1 activity prevents the up-regulation of Nox subunit gene and protein expression levels in the atherosclerotic aorta of ApoE-/- mice. (**A**–**D**) Gene expression analysis showing the suppressive effects of GSK2879552 on Nox1, Nox2, Nox4, and p22phox mRNA levels in the aorta of hypercholesterolemic ApoE -/- (HD) mice. (**E**,**F**,**I**,**J**) Western blot assay-associated densitometric analysis indicating that the relative protein abundance of each Nox subunit is significantly decreased in the aorta of GSK2879552-treated ApoE-/- (HD) mice as compared with vehicle-treated ApoE-/- (HD) mice. (**G**,**H**,**K**,**L**) Representative immunoblots showing the labeling of the Nox subunit proteins at the predicted molecular weight and the regulation of Nox subunit protein levels in the aorta of each animal group. *n* = 3–6, ** *p* < 0.01, *** *p* < 0.001. *p*-values were taken in relation to vehicle-treated ApoE-/- (ND) condition. # *p* < 0.05, ## *p* < 0.01, ### *p* < 0.001. *p*-values were taken in relation to vehicle-treated ApoE-/- (HD) condition.

Since Nox enzymes are important contributors to ROS overproduction, oxidative stress, and inflammation in atherogenesis [24,25,26,27], we analyzed the potential implication of LSD1 in the regulation of Nox subtype expression in atherosclerotic mice. By real-time PCR and Western blot assays, we detected in the atherosclerotic mice aorta significant increases in the mRNA and protein levels of Nox catalytic subunits, namely of Nox1 (mRNA: ≈ 2-fold; protein: ≈ 3-fold), Nox2 (mRNA: ≈ 3-fold; protein: ≈ 2-fold), Nox4 (mRNA: ≈ 5-fold; protein: ≈ 2-fold), and the essential subunit p22phox (mRNA: ≈ 2-fold; protein: ≈ 7-fold). Administration of GSK2879552 suppressed the up-regulation of Nox subunit expression in the aorta of atherosclerotic mice (Figure 6A–L). The data indicate that LSD1 may induce epigenetic alterations converging to up-regulation of Nox expression and, potentially, to excess formation of Nox-derived ROS in experimental atherosclerosis.

### 3.5. Inhibition of LSD1 Function Reduces the Formation of 4-HNE-Protein Adducts in the Aorta of Atherosclerotic Mice

**Figure 7:**
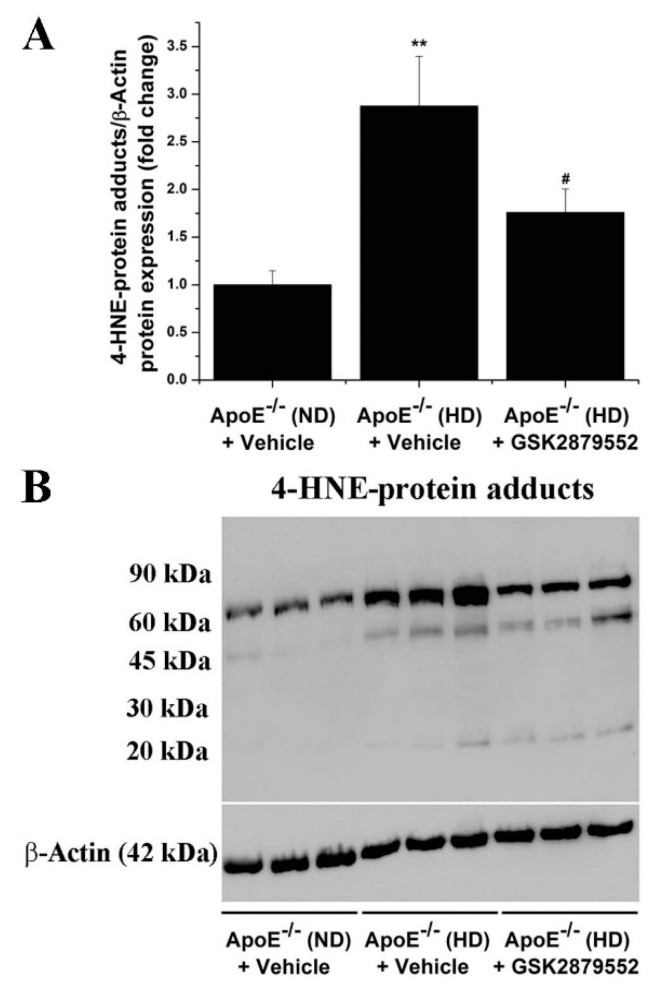
GSK2879552-induced pharmacological inhibition of LSD1 function attenuates the formation of 4-HNE-protein adducts in atherosclerotic ApoE-/- mice. (**A**) Western blot assay-related densitometric analysis showing the reduction of 4-HNE-modified protein levels in the aorta of GSK2879552-treated ApoE-/- (HD) mice. (**B**) Representative immunoblot demonstrating the inhibitory effects of GSK2879552 treatment on 4-HNE-protein adduct accumulation in the aorta of ApoE-/- mice under hypercholesterolemic conditions. *n* = 3, ** *p* < 0.01. *p*-value was taken in relation to vehicle-treated ApoE-/- (ND) condition. # *p* < 0.05. *p*-value was taken in relation to vehicle-treated ApoE-/- (HD) condition.

To investigate the impact of GSK2879552 intervention on the oxidative stress markers that indicate the sustained and excessive formation of ROS potentially generated by up-regulated Nox enzymes [28], we next examined the relative level of 4-HNE-protein adducts in the aorta of ApoE-/- mice. 4-HNE-modified proteins with molecular weights ranging from ≈90 kDa to ≈20 kDa were detected by Western blot in the aortic protein extracts. A significantly elevated formation of 4-HNE-protein adducts (≈3-fold) was found in the aorta of ApoE-/- (HD) mice compared to control mice. Long-term administration of GSK2879552 to ApoE-/- (HD) mice prevented the aortic accumulation of 4-HNE-altered proteins (Figure 7). The data suggest that LSD1-dependent signaling pathways are likely to play a role in atherogenesis by mediating ROS overproduction and the ensuing oxidative stress-induced structural alterations of proteins.

### 3.6. Pharmacological Inhibition of LSD1 Down-Regulates the Aortic Expression of Markers of Immune Cells, Inflammation, and Vascular Remodeling in Atherosclerotic ApoE-/- Mice

**Figure 8:**
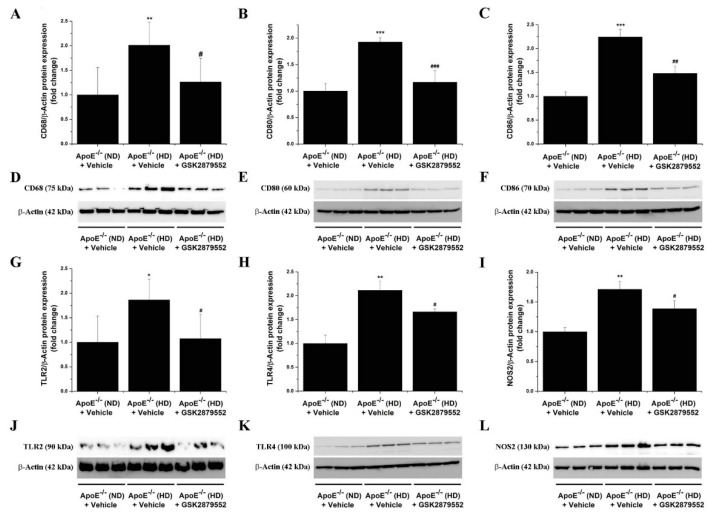
Pharmacological inhibition of LSD1 down-regulates the expression of markers of immune cells and inflammation in the atherosclerotic aorta of ApoE-/- mice. (**A**–**C**, **G**–**I**) Western blot assay-associated densitometric analysis showing the down-regulation of CD68, CD80, CD86, TLR2, TLR4, and NOS2 protein levels in the aorta of GSK2879552-treated ApoE-/- (HD) mice. (**D**–**F**, **J**–**L**) Representative immunoblots depicting the inhibitory effects of GSK2879552 treatment on the examined protein expression levels in the aorta of ApoE-/- (HD) mice. *n* = 3–6, * *p* < 0.05, ** *p* < 0.01, *** *p* < 0.001. *p*-values were taken in relation to vehicle-treated ApoE-/- (ND) condition. # *p* < 0.05, ## *p* < 0.01, ### *p* < 0.001. *p*-values were taken in relation to vehicle-treated ApoE-/- (HD) condition.

**Figure 9:**
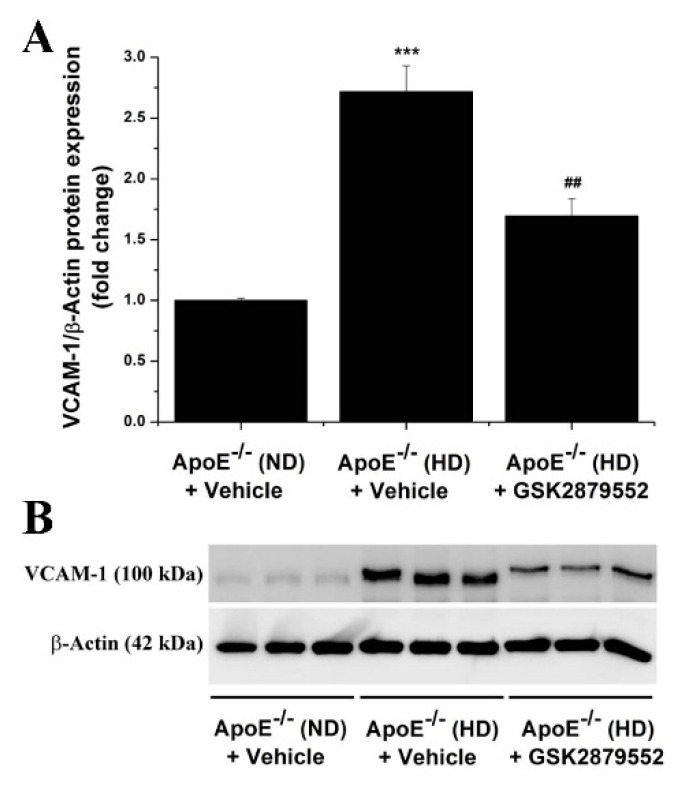
(**A**) Long-term pharmacological blockade of LSD1 function down-regulates the relative expression level of VCAM-1 protein in atherosclerotic ApoE-/- mice. (**B**) Representative immunoblot showing the modulation of aortic VCAM-1 protein level in ApoE-/- (HD) mice following GSK2879552 treatment. *n* = 3, *** *p* < 0.001. *p*-value was taken in relation to vehicle-treated ApoE-/- (ND) condition. ## *p* < 0.01. *p*-value was taken in relation to vehicle-treated ApoE-/- (HD) condition.

Based on the fact that immune cell infiltration, typically Mon/Mac in the ApoE-/- model, is instrumental for atherosclerotic lesion development [29,30], we further examined the relative expression levels of key immune cell markers in aortic homogenates derived from each animal group. As demonstrated by Western blot assays, the expression levels of CD68 (≈2-fold), CD80 (≈2-fold), CD86 (≈2-fold), TLR2 (≈1.75-fold), and TLR4 (≈2-fold) immune cell-specific proteins were found significantly augmented in the atherosclerotic aorta of ApoE-/- (HD) mice as compared with ApoE-/- (ND) mice. The molecular signature of enhanced immune cell accumulation in the atherosclerotic aorta of ApoE-/- (HD) mice was associated with a robust increase in NOS2 (≈1.75-fold) protein level, a major source of ONOO- and an indicator of inflammation. Noteworthily, up-regulated TLR2, TLR4, and NOS2 are important markers associated with a pro-inflammatory Mac phenotype [31]. Long-term administration of GSK2879552 inhibitor to ApoE-/- (HD) mice resulted in a significant attenuation in the aortic CD68, CD80, CD86, TLR2, TLR4, and NOS2 protein levels compared with vehicle-treated HD mice (Figure 8). In line with this evidence, GSK2879552 significantly diminished the up-regulation of MCP-1 (≈2-fold), TNFα (≈2.5-fold), and NOS2 (≈2.5-fold) transcript levels in the aorta of atherosclerotic mice (Figure S1 in Supplementary Materials File). Consistent with these findings, GSK2879552 intervention significantly reduced the up-regulation of VCAM-1 (≈2.5-fold), a cell surface molecule that mediates the adhesion of immune cells to vascular endothelium, in the atherosclerotic mice aorta (Figure 9). Interestingly, we detected an elevated (≈5-fold) protein level of extracellular matrix remodeling enzyme MMP9, an important trigger of plaque remodeling and destabilization, in the aorta of ApoE-/- (HD) mice that was significantly decreased by GSK2879552. However, MMP2 was not significantly down-regulated in response to LSD1 pharmacological inhibition (Figure S2 in Supplementary Materials File). Thus, other than the inhibitory effect on Nox subunit expression and attenuation of oxidative stress, GSK2879552-induced LSD1 inhibition could have an anti-atherosclerotic effect by reducing the lesional accumulation of Mon/Mac and the ensuing inflammatory response.

### 3.7. Increased Expression of LSD1 Is Associated with a Pro-Inflammatory Mac Phenotype

Since Mac infiltration is instrumental in all phases of atherosclerotic lesion development (e.g., foam cell formation, important cellular sources of ROS and inflammatory mediators), we complemented the in vivo studies with experiments on in vitro polarized pro-inflammatory (M1) and anti-inflammatory (M2) mouse Mac, an experimental set-up that recapitulated to some extent the function of different Mac populations in atheromatous lesions [31]. Significantly elevated transcript levels of LSD1/KDM1A (≈2.5-fold), KDM2A (≈3-fold), KDM3A (≈3.5-fold), KDM4A (≈3-fold), KDM5A (≈5.5-fold), and KDM5B (≈5-fold) were revealed by gene expression profiling in M1-Mac in comparison with resting (M0) Mac. The mRNA level of KDM5A was found significantly elevated (≈2.5-fold) in M2-Mac when compared with M0-Mac (Figure S3 in Supplementary Materials File). These data obtained in vitro corroborate well and extend our above results regarding KDM subtype expression in human atherosclerotic lesions and ApoE-/- mice.

### 3.8. Inhibition of LSD1 Down-Regulates the Expression of Oxidative Stress and Pro-Inflammatory Genes Associated with M1-Mac Phenotype

The observation that LSD1 is up-regulated in M1-Mac led us to hypothesize that LSD1 could play a role in shaping the Mac pro-inflammatory function. To address this issue, we examined the gene expression of Nox subtypes and key pro-inflammatory mediators in resting (M0) and polarized (M1/M2) Mac cultured in the absence/presence of 5 μM GSK2879552 for 24 h. In line with our previous study [22], significant increases in the transcript levels of the Nox subunit (Nox1 (≈20-fold), Nox2 (≈7-fold), Nox4 (≈4-fold), and p22phox (≈5-fold)) and inflammatory markers (MCP-1 (≈7-fold), TNFα (≈2.5-fold)) were determined in cultured M1-Mac. The GSK intervention resulted in a significant reduction in the transcript level of Nox subtypes and inflammatory mediators in M1-Mac. Noteworthily, LSD1 inhibition significantly attenuated the mRNA levels of Nox2, Nox4, p22phox, MCP-1, and TNFα in M0-/M2-Mac (Figures S4 and S5 in Supplementary Materials File).

### 3.9. Overexpression of LSD1 Induces the Up-Regulation of Nox Subunit Transcript Levels in HEK293 Reporter Cells

**Figure 10:**
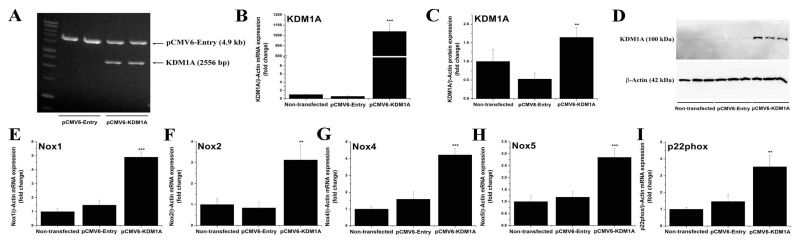
Overexpression of LSD1 triggers the up-regulation of Nox1, Nox2, Nox4, Nox5, and p22phox transcript levels in HEK293 reporter cells. (**A**) Agarose gel electrophoresis (1%) depicting the digestion products of the pCMV6-Entry and pCMV6-KDM1A expression vectors with SgfI/MluI restriction enzymes. Note the release of a ≈ 2.5 kb DNA fragment corresponding to human LSD1 (KDM1A) open reading frame following pCMV6-KDM1A enzymatic digestion. Validation of LSD1 (KDM1A) (**B**) gene and (**C**) protein up-regulation following transfection of the HEK293 cells with pCMV6-KDM1A expression vector. (**D**) Representative immunoblot depicting the up-regulation of LSD1 protein in pCMV6-KDM1A-transfected HEK293 cells. (**E**–**I**) Real-time PCR-based gene expression analysis showing the induction of Nox1, Nox2, Nox4, Nox5, and p22phox transcript levels in HEK293 cells overexpressing the human LSD1 (KDM1A) gene. *n* = 3, ** *p* < 0.01, *** *p* < 0.001. *p*-values were taken in relation to pCMV6-Entry-transfected cell condition.

To further explore the potential implication of LSD1 in mediating Nox up-regulation, we performed transfection assays employing HEK293 reporter cells to overexpress the human LSD1 gene. This is an artificial experimental set-up that mimics, to some extent, the up-regulation of LSD1 associated with both human and experimental atherosclerosis, namely atherosclerotic ApoE-/- mice and cultured pro-inflammatory (M1) Mac. Noteworthily, previous studies showed that the HEK293 cell line represents a reliable in vitro model to investigate various aspects of Nox regulation and function [32,33,34]. Transient transfection of HEK293 cells with pCMV6-KDM1A resulted in a marked up-regulation of LSD1/KDM1A mRNA (≈1000-fold) and protein (≈3-fold) levels. Of note, significant increases in Nox1 (≈5-fold), Nox2 (≈3-fold), Nox4 (≈4-fold), Nox5 (≈3-fold), and p22phox (≈3-fold) transcript levels were detected in HEK293 reporter cells overexpressing LSD1/KDM1A as compared with empty vector (pCMV6-Entry)-transfected cells (Figure 10). These results further strengthen the hypothesis whereby up-regulated LSD1 triggers a chain of molecular events converging to enhanced expression of Nox subtypes in experimental atherosclerosis.

## 4. Discussion

As mentioned above, emerging clinical and experimental evidence indicates that epigenetic instability is mechanistically associated with transcriptomic alterations in CVD. Over time, these alterations could lead to the imprinting of a long-lasting toxic epigenetic memory, a condition that may further aggravate the oxidative reactions and inflammatory responses in the vasculature [4,35,36,37,38,39,40].

The abnormal expression pattern of selective histone methylation marks, particularly enhanced H3K4me2 and reduced H3K9me2 levels, was determined in endothelial cells, vascular smooth muscle cells, and infiltrated inflammatory immune cells within human advanced atherosclerotic lesions [14]. These important findings potentially implicate LSD1 in the process of atheroma formation, among other dysregulated KMT and KDM subtypes. Nonetheless, the role of histone methylation-based pathways and in particular LSD1 in atherosclerosis is still incompletely understood, regardless of major breakthroughs in the field of epigenetics of CVD [41].

Intrigued by this fact, we questioned whether LSD1 could control important pathological networks and downstream molecular effectors linked to the regulation of cellular sources of ROS overproduction (i.e., Nox enzymes), oxidative stress-induced structural alterations of proteins, and inflammatory markers in atherosclerosis.

To address this issue, we employed (i) human non-atherosclerotic and atherosclerotic tissue specimens, (ii) in vivo experimental model of atherosclerosis, namely ApoE-/- mice, and (iii) in vitro polarized M1/M2-Mac derived from primary cultures of mouse Mon.

The key findings of this study are as follows: (1) the expression levels of archetypal KDM subtypes, including KDM1A (LSD1), KDM2A, KDM3A, KDM4A, KDM5A, and KDM5B, are significantly elevated in human atheromas, aortas of atherosclerotic mice, and cultured pro-inflammatory Mac; (2) long-term pharmacological inhibition of LSD1 reduces the formation of atherosclerotic lesions in hypercholesterolemic ApoE-/- mice; (3) LSD1 is implicated in or mediates the up-regulation of mRNA and protein expression levels of Nox1, Nox2, Nox4, and p22phox and the formation of 4-HNE-protein adducts in the aorta of atherosclerotic mice, as demonstrated by the inhibitory effect of GSK2879552 treatment; (4) blockade of LSD1 function significantly reduces the aortic expression of markers of immune cell infiltration, inflammation, and vascular remodeling (CD68, CD80, CD86, TLR2, TLR4, NOS2, VCAM-1, MMP9) in atherosclerotic mice; (5) inhibition of LSD1 suppresses the up-regulation of genes mechanistically associated with oxidative stress (Nox1, Nox2, Nox4, p22phox) and inflammation (MCP-1, TNFα) in cultured mouse pro-inflammatory Mac; (6) overexpression of LSD1/KDM1A induces Nox1, Nox2, Nox4, Nox5, and p22phox transcript levels in HEK293 reporter cells.

In search of the prospective implication of selective KDMs in atherosclerosis, we performed initial gene/protein expression profiling of archetypal KDM subtypes in atherosclerotic plaques derived from patients with severe carotid artery stenosis. The results showed significant increases in LSD1 (KDM1A) mRNA and protein levels, along with other KDMs heaving a steady or significantly up-regulated expression in advanced human atherosclerotic lesions as compared with non-atherosclerotic tissue specimens.

To gain pathophysiological and mechanistic insights into the role of LSD1 in atherogenesis, ApoE-/- mice were employed as an in vivo model of atherosclerosis. Significantly elevated mRNA and protein levels of LSD1/KDM1A, along with up-regulated transcript levels of KDM2A, KDM3A, KDM4A, KDM5A, and KDM5B subtypes, were determined in the aorta of ApoE-/- mice with established atherosclerosis, an experimental set-up that recapitulated to some extent important pathophysiological characteristics of human atherosclerotic disease [19]. Considering the highly similar gene and/or protein expression pattern of selective KDMs, including LSD1, in both clinical and experimental atherosclerosis, we may speculate that these epigenetic enzymes direct, at least in part, comparable pathobiological effects underlying atheroma formation in humans and ApoE-/- mice.

Clinical and experimental oncology provided much of the current understanding of LSD1 expression and function. Mechanistically, the up-regulation of LSD1 in carcinogenesis induces the demethylation of H3K4me1/2 and, consequently, the transcriptional repression of specific genes contributing to various aspects of human malignancies, such as alteration of tumor suppression mechanisms, growth arrest, cell differentiation, and apoptosis [42,43]. Despite being clinically different diseases, various forms of cancer and atherosclerosis display, to some extent, overlapping dysregulated molecular effectors and signaling pathways typically associated with exacerbation of ROS production, oxidative stress, inflammation, cellular phenotypic alterations, and enhanced cell proliferation and migration. In this context, further assessment of clinically approved compounds for various pathologies (i.e., cancer) may be considered for drug repurposing for related pharmacological targets in atherosclerosis. Such a strategy could potentially accelerate the development of novel treatment algorithms to improve the therapeutic outcome in CVD. Therefore, we tested a potent, highly specific, and clinically approved LSD1 pharmacological inhibitor developed by the GlaxoSmithKline Company (GSK2879552) for cancer therapy, particularly for acute myeloid leukemia (AML) and small lung cancer cells (SCLC) [44]. Our study provides evidence that GSK2879552-induced inhibition of LSD1 significantly reduces the progression of atherosclerotic lesions in hypercholesterolemic mice without having a significant impact on plasma levels of total cholesterol and triglycerides.

Since ROS overproduction, typically generated by up-regulated Nox, is acknowledged as a key trigger of redox-sensitive pro-inflammatory signaling pathways and oxidative stress-induced insults in atherosclerosis [24,25,26,27], we next investigated the pathophysiological scenario whereby dysregulated LSD1 plays a role in the modulation of Nox subunit gene and protein expression in atherosclerotic mice. We provide evidence that the pharmacological blockade of LSD1 suppresses the up-regulation of mRNA and protein levels of Nox catalytic subunits, namely Nox1, Nox2, and Nox4, as well as of the p22phox essential subunit in the atherosclerotic mice aorta. Noteworthily, it has been previously demonstrated that pharmacological inhibition of Nox1/4 function (GKT137831) or genetic ablation of various Nox subtypes (e.g., p47phox, Nox2) reduces ROS overproduction and the atherosclerotic lesion formation in ApoE-/- mice [25,26,27]. Considering this convincing evidence and our results, we may assume that the anti-atherosclerotic effects of GSK2879552 treatment are likely to be determined, at least in part, by the down-regulation of Nox expression and the ensuing reduced Nox-derived ROS formation in the aorta of ApoE-/- mice.

As reported by several comprehensive studies, the up-regulation of Nox subunit expression is typically associated with an overall increase in NADPH-stimulated ROS production in different cardiovascular disorders [27]. Yet, considering the relative specificity of the currently used methods to assess ROS production, the implication of other sources should be considered [45,46]. Thus, the detection of ROS production reflecting the highly dynamic and complex in vivo conditions represents a major challenge in free radical biology research [47]. Considering these technical aspects, instead of measuring the potential of aortic tissue to produce ROS in a test tube, we indirectly assessed the formation of ROS in a pathologically relevant context. Covalent modification of proteins at histidine residues by 4-HNE resulting in 4-HNE-protein adduct formation is acknowledged as a reliable and long-lasting marker for oxidative stress insult in different pathologies. Thus, we determined the relative level of the oxidative stress-induced structural alteration of proteins, namely the aortic formation of 4-HNE-protein adducts in mice. Interestingly, the levels of 4-HNE-protein adducts were significantly reduced in the aorta of atherosclerotic mice following GSK treatment. These data further support the implication of LSD1 in atherogenesis by regulating target genes (e.g., Nox) and signaling pathways converging to ROS overproduction and oxidative stress. Noteworthily, it has been shown that ROS production driven by activated Nox contributes to 4-HNE formation in experimental models of cardiovascular diseases [28].

Possible mechanistic explanations regarding LSD1-induced Nox subunit up-regulation may emerge from previous studies demonstrating that advanced human atherosclerotic lesions are associated with decreased levels of H3K9me2, an important histone substrate of LSD1 demethylation activity [14]. Thus, we may speculate that the up-regulation of LSD1 in atherosclerosis functions as a key epigenetic inducer of gene transcription by reducing the level of repressive histone marks. In line with this hypothesis, we demonstrate in this study that transient overexpression of LSD1 in HEK293 reporter cells produces significant increases in human Nox1, Nox2, Nox4, Nox5, and p22phox transcript levels. This evidence suggests that Nox subunit genes may be important targets of LSD1, a key finding that further implicates LSD1-dependent signaling pathways not only in atherogenesis, but also in other different pathologies associated with increased Nox expression, ROS overproduction, and oxidative stress (e.g., cancer, diabetes, hypertension, neurodegeneration).

Other than H3K4/H3K9 histones, LSD1 regulates the function of multiple non-histone proteins, including transcription factors [13]. Evidence exists that LSD1 plays a major role in mediating inflammatory response in various cell types, by molecular mechanisms that involve the activation of the NF-kB transcription factor [48,49,50]. Since NF-kB is an important transcriptional regulator of Nox subtypes in vascular and immune cells under pro-inflammatory conditions [51,52,53], we may safely assume that LSD1 plays a role in the regulation of Nox subunit expression via NF-kB-dependent mechanisms. Furthermore, a highly organized functional crosstalk between LSD1 and HDAC1 has been demonstrated in breast cancer cells, in which silencing of LSD1 expression augmented the tumor suppressive effects of the pan-HDAC inhibitor SAHA [54,55,56]. Consistent with this important evidence highlighting that some HDAC inhibitors may affect LSD1 activity, we have previously demonstrated that pan-HDAC inhibition by SAHA reduces Nox expression, oxidative stress, the level of immune cell markers, and the progression of atherosclerotic lesions in the aorta of hypercholesterolemic ApoE-/- mice [22,57]. It is important to note that the GSK-induced inhibition of LSD1 also produced significant decreases in the expression of markers of immune cell infiltration (CD68, CD80, CD86, TLR2, TLR4), inflammation (VCAM-1, NOS2), and vascular remodeling (MMP9) in the aorta of atherosclerotic mice. Collectively, these data further implicate LSD1 in the etiology and progression of atherosclerotic lesions and provide additional support for a potential LSD1-HDAC1 functional interplay in atherogenesis.

Since Mon-derived Mac are important cellular drivers of atherosclerotic lesion formation [31], we examined the potential implication of LSD1 in mediating the Mac inflammatory response. For this purpose, in vitro polarized pro-inflammatory (M1) and anti-inflammatory (M2) mouse Mac were employed. The transcript levels of LSD1 (KDM1A), KDM2A, KDM3A, KDM4A, KDM5A, and KDM5B were found significantly elevated in cultured M1-Mac in a highly similar manner with the KDM subtype expression pattern associated with both human and mouse atherosclerotic lesions. Moreover, inhibition of LSD1 significantly reduced the transcript levels of important markers associated with M1-Mac phenotype, namely, Nox subtypes, MCP-1, and TNFα. Considering the fact that LPS is a key inducer of M1-Mac polarization in vitro, our data are in a good agreement and extend a previous study demonstrating that LSD1 plays a major role in mediating LPS-induced inflammatory response of vascular smooth muscle cells via NF-kB-dependent signaling. Consistent with these findings, it has been demonstrated that LSD1 is a trigger of renal inflammation in hepatitis B virus-associated glomerulonephritis [58]. Collectively, these data strengthen the role LSD1 as an important regulator of inflammatory signaling pathways in different pathological states.

It is worth mentioning that dysfunctional endothelial cells (ECs) and vascular smooth muscle cells (VSMCs) are instrumental in the process of atheroma formation. Interestingly, pharmacological inhibition of LSD1 reduced EC proliferation and inflammatory response, VSMC proliferation, and neointimal hyperplasia [59,60,61]. Together, these data suggest that LSD1-related signaling pathways could regulate important pathological aspects in multiple cell types implicated in atherogenesis.

Besides oxidative stress and inflammation, alterations in lipid metabolism play a major role in atherogenesis. Reportedly, LSD1 stimulates lipogenesis by acting as a positive regulator of sterol regulatory element-binding proteins, whilst knockdown of LSD1 decreases triglyceride levels in hepatocytes [62]. In the line with this evidence, it was shown that inhibition of LSD1 prevents metabolic alterations in obese db/db mice by increasing insulin sensitivity in the adipose tissue and reducing triglyceride accumulation and gluconeogenesis in the liver [63]. Collectively, these reports indicate that LSD1 controls important pathways in lipid metabolism. Yet, the potential mechanistic links between dysregulated LSD1 and the modulation of cholesterol transport pathways and the foam cell formation process in atherosclerosis remain open issues and require further attention.

Noteworthily, in addition to LSD1 (KDM1A), other archetypal KDM subtypes were found significantly up-regulated in human and experimental atherosclerosis. As a function of the pathophysiological context, some of these enzymes display similar activities in the regulation of gene expression whilst other KDMs possess opposite biological functions [5]. Thus, it was previously demonstrated that KDM3A plays a major role in the up-regulation of Nox2 and Nox4 expression in experimental myocardial infarction [64]. Other than that, KDM3A has been identified as important regulator of neointima formation in experimental atherosclerosis [65] whilst KDM4A mediates oxidized LDL-induced pro-inflammatory Mac polarization [66]. Furthermore, members of the KDM5 histone demethylase family were associated with CVD [67]. Noteworthily, KDM5B has been implicated in the up-regulation of NOS2 expression [68,69]. Moreover, unveiling the specific roles of KMT-dependent signaling pathways in atherosclerotic lesion formation remains an open issue that should be further addressed.

Consequently, the existence of positive or negative feedback regulatory mechanisms within the KDM family and the complex networking and functional cooperation between KDMs, KMTs, and gene-specific transcription factors remain arguable issues. In addition, the limitations of the ApoE-/- animal model, and animal models of atherosclerosis in general, in accurately replicating the complexity of severe human atherosclerosis as well as the potential occurrence of gender-specific epigenetic alterations should be considered.

## 5. Conclusions

In conclusion, the novelty of this study is the demonstration that LSD1 is up-regulated in human advanced atherosclerotic lesions, as well as in in vivo and in vitro experimental models of atherosclerosis. Importantly, LSD1 inhibition reduces the progression of atherosclerotic lesions by a mechanism that involves the down-regulation of the expression of key molecular effectors contributing to atheroma formation in the aorta of hypercholesterolemic mice. This mechanism is the negative regulation of Nox subunit expression and potentially ROS overproduction as reflected in the reduced level of oxidative stress-induced structural alterations of proteins, mitigation of immune cell infiltration, inflammation, and vascular remodeling markers. Nevertheless, other pathophysiological processes that could be influenced by LSD1 inhibition remain to be further examined. Collectively, the data suggest that LSD1 is a novel therapeutic target and that LSD1-oriented pharmacological interventions could become an important supportive therapeutic strategy in atherosclerosis-related CVD.

## Data Availability

Data are contained within the article or Supplementary Materials.

## Supplementary Materials

The following supporting information can be downloaded at: https://www.mdpi.com/article/10.3390/antiox11122382/s1, Table S1. Clinical characteristics of the patients. Table S2: Sequences of sense and antisense oligonucleotide primers used in real-time PCR assays; Figure S1: LSD1 blockade suppresses the up-regulation of MCP-1, TNFα, and NOS2 transcript levels in the atherosclerotic aorta of ApoE-/- mice; Figure S2: LSD1 mediates the up-regulation of MMP9 but not MMP2 protein levels in the atherosclerotic aorta of ApoE-/- mice; Figure S3: Elevated mRNA expression levels of LSD1/KDM1A, KDM2A, KDM3A, KDM4A, KDM5A, and KDM5B subtypes are associated with a pro-inflammatory macrophage phenotype in vitro; Figure S4: GSK2879552-induced pharmacological inhibition of LSD1 function suppresses the up-regulation of Nox1, Nox2, Nox4, and p22phox mRNA levels in cultured pro-inflammatory macrophages; Figure S5: LSD1 blockade reduces the up-regulation of MCP-1 and TNFα transcript levels in cultured pro-inflammatory macrophages.

## References

[1] Zhang W., Song M., Qu J., Liu G.H. (2018). Epigenetic modifications in cardiovascular aging and diseases. Circ. Res..

[2] Wang Y., Miao X., Liu Y., Li F., Liu Q., Sun J., Cai L. (2014). Dysregulation of histone acetyltransferases and deacetylases in cardiovascular diseases. Oxidative Med. Cell. Longev..

[3] Herman A.B., Occean J.R., Sen P. (2021). Epigenetic dysregulation in cardiovascular aging and disease. J. Cardiovasc. Aging..

[4] Costantino S., Libby P., Kishore R., Tardif J.C., El-Osta A., Paneni F. (2018). Epigenetics and precision medicine in cardiovascular patients: From basic concepts to the clinical arena. Eur. Heart J..

[5] Morera L., Lübbert M., Jung M. (2016). Targeting histone methyltransferases and demethylases in clinical trials for cancer therapy. Clin. Epigenetics.

[6] Jiang W., Agrawal D.K., Boosani C.S. (2018). Cell specific histone modifications in atherosclerosis. Mol. Med. Rep..

[7] Wei X., Yi X., Zhu X.H., Jiang D.S. (2020). Histone methylation and vascular biology. Clin. Epigenetics.

[8] Lin Y., Qiu T., Wei G., Que Y., Wang W., Kong Y., Xie T., Chen X. (2022). Role of histone post-translational modifications in inflammatory diseases. Front. Immunol..

[9] Yun M., Wu J., Workman J.L., Li B. (2011). Readers of histone modifications. Cell. Res..

[10] Kim D., Kim K.I., Baek S.H. (2021). Roles of lysine-specific demethylase 1 (LSD1) in homeostasis and diseases. J. Biomed. Sci..

[11] Gu F., Lin Y., Wang Z., Wu X., Ye Z., Wang Y., Lan H. (2020). Biological roles of LSD1 beyond its demethylase activity. Cell. Mol. Life Sci..

[12] Lan H., Tan M., Zhang Q., Yang F., Wang S., Li H., Xiong X., Sun Y. (2019). LSD1 destabilizes FBXW7 and abrogates FBXW7 functions independent of its demethylase activity. Proc. Natl. Acad. Sci. USA.

[13] Perillo B., Tramontano A., Pezone A., Migliaccio A. (2020). LSD1: More than demethylation of histone lysine residues. Exp. Mol. Med..

[14] Greißel A., Culmes M., Burgkart R., Zimmermann A., Eckstein H.H., Zernecke A., Pelisek J. (2016). Histone acetylation and methylation significantly change with severity of atherosclerosis in human carotid plaques. Cardiovasc. Pathol..

[15] Harman J.L., Dobnikar L., Chappell J., Stokell B.G., Dalby A., Foote K., Finigan A., Freire-Pritchett P., Taylor A.L., Worssam M.D. (2019). Epigenetic regulation of vascular smooth muscle cells by histone H3 lysine 9 dimethylation attenuates target gene-induction by inflammatory Signaling. Arterioscler. Thromb. Vasc. Biol..

[16] Yang Y., Luan Y., Yuan R.X., Luan Y. (2021). Histone methylation related therapeutic challenge in cardiovascular diseases. Front. Cardiovasc. Med..

[17] Kietzmann T., Petry A., Shvetsova A., Gerhold J.M., Görlach A. (2017). The epigenetic landscape related to reactive oxygen species formation in the cardiovascular system. Br. J. Pharmacol..

[18] Yi X., Zhu Q.X., Wu X.L., Tan T.T., Jiang X.J. (2022). Histone methylation and oxidative stress in cardiovascular diseases. Oxid. Med. Cell. Longev..

[19] Nakashima Y., Plump A.S., Raines E.W., Breslow J.L., Ross R. (1994). ApoE-deficient mice develop lesions of all phases of atherosclerosis throughout the arterial tree. Arterioscler. Thromb..

[20] Mohammad H.P., Smitheman K.N., Kamat C.D., Soong D., Federowicz K.E., Van Aller G.S., Schneck J.L., Carson J.D., Liu Y., Butticello M. (2015). A DNA hypomethylation signature predicts antitumor activity of LSD1 inhibitors in SCLC. Cancer Cell..

[21] Macheleidt I.F., Dalvi P.S., Lim S.Y., Meemboor S., Meder L., Käsgen O., Müller M., Kleemann K., Wang L., Nürnberg P. (2018). Preclinical studies reveal that LSD1 inhibition results in tumor growth arrest in lung adenocarcinoma independently of driver mutations. Mol. Oncol..

[22] Manea S.A., Vlad M.L., Fenyo I.M., Lazar A.G., Raicu M., Muresian H., Simionescu M., Manea A. (2020). Pharmacological inhibition of histone deacetylase reduces NADPH oxidase expression, oxidative stress and the progression of atherosclerotic lesions in hypercholesterolemic apolipoprotein E-deficient mice; potential implications for human atherosclerosis. Redox Biol..

[23] Pfaffl M.W. (2001). A new mathematical model for relative quantification in real-time RT-PCR. Nucleic Acids Res..

[24] Buchmann G.K., Schürmann C., Warwick T., Schulz M.H., Spaeth M., Müller O.J., Schröder K., Jo H., Weissmann N., Brandes R.P. (2020). Deletion of NoxO1 limits atherosclerosis development in female mice. Redox Biol..

[25] Gray S.P., Di Marco E., Okabe J., Szyndralewiez C., Heitz F., Montezano A.C., de Haan J.B., Koulis C., El-Osta A., Andrews K.L. (2013). NADPH oxidase 1 plays a key role in diabetes mellitus-accelerated atherosclerosis. Circulation.

[26] Gray S.P., Jha J.C., Kennedy K., van Bommel E., Chew P., Szyndralewiez C., Touyz R.M., Schmidt H.H.H.W., Cooper M.E., Jandeleit-Dahm K.A.M. (2017). Combined NOX1/4 inhibition with GKT137831 in mice provides dose-dependent reno- and atheroprotection even in established micro- and macrovascular disease. Diabetologia.

[27] Sirker A., Zhang M., Shah A.M. (2011). NADPH oxidases in cardiovascular disease: Insights from in vivo models and clinical studies. Basic Res. Cardiol..

[28] Manea A., Manea S.A., Todirita A., Albulescu I.C., Raicu M., Sasson S., Simionescu M. (2015). High-glucose-increased expression and activation of NADPH oxidase in human vascular smooth muscle cells is mediated by 4-hydroxynonenal-activated PPARα and PPARβ/δ. Cell Tissue Res..

[29] Kuznetsova T., Prange K.H.M., Glass C.K., de Winther M.P.J. (2020). Transcriptional and epigenetic regulation of macrophages in atherosclerosis. Nat. Rev. Cardiol..

[30] Hoeksema M.A., de Winther M.P. (2016). Epigenetic regulation of monocyte and macrophage function. Antioxid. Redox Signal..

[31] Chinetti-Gbaguidi G., Colin S., Staels B. (2015). Macrophage subsets in atherosclerosis. Nat. Rev. Cardiol..

[32] Kawahara T., Ritsick D., Cheng G., Lambeth J.D. (2005). Point mutations in the proline-rich region of p22phox are dominant inhibitors of Nox1- and Nox2-dependent reactive oxygen generation. J. Biol. Chem..

[33] Sweeny E.A., Schlanger S., Stuehr D.J. (2020). Dynamic regulation of NADPH oxidase 5 by intracellular heme levels and cellular chaperones. Redox Biol..

[34] Han J., Jin C., Zhong Y., Zhu J., Liu Q., Sun D., Feng J., Xia X., Peng X. (2021). Involvement of NADPH oxidase in patulin-induced oxidative damage and cytotoxicity in HEK293 cells. Food Chem. Toxicol..

[35] Wierda R.J., Geutskens S.B., Jukema J.W., Quax P.H., van den Elsen P.J. (2010). Epigenetics in atherosclerosis and inflammation. J. Cell. Mol. Med..

[36] Villeneuve L.M., Reddy M.A., Lanting L.L., Wang M., Meng L., Natarajan R. (2008). Epigenetic histone H3 lysine 9 methylation in metabolic memory and inflammatory phenotype of vascular smooth muscle cells in diabetes. Proc. Natl. Acad. Sci. USA.

[37] Reddy M.A., Natarajan R. (2011). Epigenetic mechanisms in diabetic vascular complications. Cardiovasc. Res..

[38] Gomez D., Swiatlowska P., Owens G.K. (2015). Epigenetic control of smooth muscle cell identity and lineage memory. Arterioscler. Thromb. Vasc. Biol..

[39] Chelladurai P., Seeger W., Pullamsetti S.S. (2016). Epigenetic mechanisms in pulmonary arterial hypertension: The need for global perspectives. Eur. Respir. Rev..

[40] Edgar L., Akbar N., Braithwaite A.T., Krausgruber T., Gallart-Ayala H., Bailey J., Corbin A.L., Khoyratty T.E., Chai J.T., Alkhalil M. (2021). Hyperglycemia induces trained immunity in macrophages and their precursors and promotes atherosclerosis. Circulation.

[41] Arifuzzaman S., Khatun M.R., Khatun R. (2020). Emerging of lysine demethylases (KDMs): From pathophysiological insights to novel therapeutic opportunities. Biomed. Pharmacother..

[42] Zhang J., Zhao D., Li Q., Du X., Liu Y., Dai X., Hong L. (2019). Upregulation of LSD1 promotes migration and invasion in gastric cancer through facilitating EMT. Cancer Manag. Res..

[43] Karakaidos P., Verigos J., Magklara A. (2019). LSD1/KDM1A, a gate-keeper of cancer stemness and a promising therapeutic target. Cancers (Basel).

[44] Fang Y., Liao G., Yu B. (2019). LSD1/KDM1A inhibitors in clinical trials: Advances and prospects. J. Hematol. Oncol..

[45] Rezende F., Löwe O., Helfinger V., Prior K.K., Walter M., Zukunft S., Fleming I., Weissmann N., Brandes R.P., Schröder K. (2016). Unchanged NADPH oxidase activity in Nox1-Nox2-Nox4 triple knockout mice: What do NADPH-stimulated chemiluminescence assays really detect?. Antioxid. Redox Signal..

[46] Brandes R.P., Rezende F., Schröder K. (2018). Redox regulation beyond ROS: Why ROS should not be measured as often. Circ. Res..

[47] Manea S.A., Vlad M.L., Rebleanu D., Lazar A.G., Fenyo I.M., Calin M., Simionescu M., Manea A. (2021). Detection of vascular reactive oxygen species in experimental atherosclerosis by high-resolution near-infrared fluorescence imaging using VCAM-1-targeted liposomes entrapping a fluorogenic redox-sensitive probe. Oxid. Med. Cell. Longev..

[48] Kim D., Nam H.J., Lee W., Yim H.Y., Ahn J.Y., Park S.W., Shin H.J.R., Yu R., Won K.J., Bae J.S. (2018). PKCα-LSD1-NF-κB-signaling cascade is crucial for epigenetic control of the inflammatory response. Mol. Cell..

[49] Haines R.R., Scharer C.D., Lobby J.L., Boss J.M. (2019). LSD1 cooperates with noncanonical NF-κB signaling to regulate marginal zone B cell development. J. Immunol..

[50] Liang L., Sun M., Qi Z., Li W. (2020). Lysine demethylase 1A exacerbates LPS-induced inflammation of vascular smooth muscle cells through modulation of NF-κB activation. Trop. J. Pharm. Res..

[51] Anrather J., Racchumi G., Iadecola C. (2006). NF-kappaB regulates phagocytic NADPH oxidase by inducing the expression of gp91phox. J. Biol. Chem..

[52] Manea A., Manea S.A., Gafencu A.V., Raicu M. (2007). Regulation of NADPH oxidase subunit p22(phox) by NF-kB in human aortic smooth muscle cells. Arch. Physiol. Biochem..

[53] Manea A., Tanase L.I., Raicu M., Simionescu M. (2010). Transcriptional regulation of NADPH oxidase isoforms, Nox1 and Nox4, by nuclear factor-kappaB in human aortic smooth muscle cells. Biochem. Biophys. Res. Commun..

[54] Huang Y., Vasilatos S.N., Boric L., Shaw P.G., Davidson N.E. (2012). Inhibitors of histone demethylation and histone deacetylation cooperate in regulating gene expression and inhibiting growth in human breast cancer cells. Breast Cancer Res. Treat..

[55] Vasilatos S.N., Katz T.A., Oesterreich S., Wan Y., Davidson N.E., Huang Y. (2013). Crosstalk between lysine-specific demethylase 1 (LSD1) and histone deacetylases mediates antineoplastic efficacy of HDAC inhibitors in human breast cancer cells. Carcinogenesis.

[56] Nalawansha D.A., Pflum M.K.H. (2017). LSD1 substrate binding and gene expression are affected by HDAC1-mediated deacetylation. ACS Chem. Biol..

[57] Manea S.A., Antonescu M.L., Fenyo I.M., Raicu M., Simionescu M., Manea A. (2018). Epigenetic regulation of vascular NADPH oxidase expression and reactive oxygen species production by histone deacetylase-dependent mechanisms in experimental diabetes. Redox Biol..

[58] Yang Y.T., Wang X., Zhang Y.Y., Yuan W.J. (2019). The histone demethylase LSD1 promotes renal inflammation by mediating TLR4 signaling in hepatitis B virus-associated glomerulonephritis. Cell Death Dis..

[59] Wojtala M., Rybaczek D., Wielgus E., Sobalska-Kwapis M., Strapagiel D., Balcerczyk A. (2021). The role of lysine-specific demethylase 1 (LSD1) in shaping the endothelial inflammatory response. Cell. Physiol. Biochem..

[60] Zhang X., Huang T., Zhai H., Peng W., Zhou Y., Li Q., Yang H. (2020). Inhibition of lysine-specific demethylase 1A suppresses neointimal hyperplasia by targeting bone morphogenetic protein 2 and mediating vascular smooth muscle cell phenotype. Cell Prolif..

[61] Yuan B., Liu H., Pan X., Dong X., Qu L.F., Sun J., Pan L.L. (2022). LSD1 downregulates p21 expression in vascular smooth muscle cells and promotes neointima formation. Biochem. Pharmacol..

[62] Abdulla A., Zhang Y., Hsu F.N., Xiaoli A.M., Zhao X., Yang E.S.T., Ji J.Y., Yang F. (2014). Regulation of lipogenic gene expression by lysine-specific histone demethylase-1 (LSD1). J. Biol. Chem..

[63] Ramms B., Pollow D.P., Zhu H., Nora C., Harrington A.R., Omar I., Gordts P.L.S.M., Wortham M., Sander M. (2022). Systemic LSD1 inhibition prevents aberrant remodeling of metabolism in obesity. Diabetes.

[64] Li Z., Zhang X., Liu S., Zeng S., Yu L., Yang G., Guo J., Xu Y. (2018). BRG1 regulates NOX gene transcription in endothelial cells and contributes to cardiac ischemia-reperfusion injury. Biochim. Biophys. Acta Mol. Basis Dis..

[65] Chen J., Zhang J., Yang J., Xu L., Hu Q., Xu C., Yang S., Jiang H. (2017). Histone demethylase KDM3a, a novel regulator of vascular smooth muscle cells, controls vascular neointimal hyperplasia in diabetic rats. Atherosclerosis.

[66] Wang X., Wang S., Yao G., Yu D., Chen K., Tong Q., Ye L., Wu C., Sun Y., Li H. (2017). Identification of the histone lysine demethylase KDM4A/JMJD2A as a novel epigenetic target in M1 macrophage polarization induced by oxidized LDL. Oncotarget.

[67] Mokou M., Klein J., Makridakis M., Bitsika V., Bascands J.L., Saulnier-Blache J.S., Mullen W., Sacherer M., Zoidakis J., Pieske B. (2019). Proteomics based identification of KDM5 histone demethylases associated with cardiovascular disease. EBioMedicine.

[68] Han M., Xu W., Cheng P., Jin H., Wang X. (2017). Histone demethylase lysine demethylase 5B in development and cancer. Oncotarget.

[69] Rao C.V., Kawamori T., Hamid R., Reddy B.S. (1999). Chemoprevention of colonic aberrant crypt foci by an inducible nitric oxide synthase-selective inhibitor. Carcinogenesis.

