# Supplementary material for: Pharmacological Inhibition of Lysine-Specific Demethylase 1A Reduces Atherosclerotic Lesion Formation in Apolipoprotein E-Deficient Mice by a Mechanism Involving Decreased Oxidative Stress and Inflammation; Potential Implications in Human Atherosclerosis
Source: Antioxidants (Basel). 2022 Dec 1;11(12):2382. doi: 10.3390/antiox11122382 (PMC9774905; doi:10.3390/antiox11122382)
Supplement: Supplementary file 1 [file antioxidants-11-02382-s001.zip › antioxidants-2046608-supplementary/Supplementary Materials file_Antioxidants 2022_Revision.pdf]

Supplementary Materials file

Pharmacological inhibition of lysine-specific demethylase 1A reduces atherosclerotic lesion formation in apolipoprotein E-deficient mice by a mechanism involving decreased oxidative stress and inflammation; evidence for potential implications in human atherosclerosis

Simona-Adriana Manea[1#], Mihaela-Loredana Vlad[1#], Alexandra-Gela Lazar[1], Horia Muresian[2], Maya Simionescu[1], Adrian Manea[1*]

[1] Institute of Cellular Biology and Pathology "Nicolae Simionescu" of the Romanian Academy, Bucharest, Romania

[2] University Hospital Bucharest, Cardiovascular Surgery Department, Bucharest, Romania

#

These authors contributed equally to this work

*

Corresponding author:

Adrian Manea, Ph.D.

e-mail: adrian.manea@icbp.ro

Institute of Cellular Biology and Pathology "Nicolae Simionescu" of the Romanian Academy, 8, B.P. Hasdeu Street, 050568, Bucharest, Romania

Parameter	Values (n = 25)
Gender: Female (F)/Male (M) (N, %)	9/16 (36%/64%)
Age (years)	64.2 ± 8.3
Body-mass index (BMI) (kg/m2)	26.1 ± 3.6
Current smoker (N, %)	16 (64%)
Former smoker (N, %)	4 (16%)
Alcohol consumption (N, %)	1 (4%)
Diabetes (N, %)	8 (32%)
Hypertension (N, %)	21 (84%)
**Other cardiac diseases**	
Coronary artery disease (CAD) (N, %)	9 (36%)
Peripheral artery disease (PAD) (N, %)	8 (32%)
Glucose (mg/dl)	110.8 ± 33.6
TC (mg/dl)	175.1 ± 59.4
CHOL LDL (mg/dl)	116.8 ± 50.2
CHOL HDL (mg/dl)	43.5 ± 11.3
TG (mg/dl)	120 ± 46.2
**Medication**	
Aspirin (N, %)	23 (92 %)
Insulin (N, %)	1 (4%)
Metformin (N, %)	7 (28%)
Sulphonylurea (N, %)	2 (8 %)
Statin (N, %)	24 (96 %)

Table S1. Clinical characteristics of the patients. TC: total cholesterol; CHOL LDL: cholesterol low-density lipoprotein fraction; CHOL HDL: cholesterol high-density lipoprotein fraction; TG: triglycerides.

Gene	GeneBank® Accession Number	Sequences of oligonucleotide primers
**Mouse Nox1**	NM_172203.2	S: 5'-CATCCAGTCTCCAAACATGACA-3'<br>A: 5'-GCTACAGTGGCAATCACTCCAG-3'
**Mouse Nox2**	FJ168469.1	S: 5'-ACTCCTTGGGTCAGCACTGG-3'<br>A: 5'-GTTCCTGTCCAGTTGTCTTCG-3'
**Mouse Nox4**	AF276957.1	S: 5'-TGAACTACAGTGAAGATTTCCTTGAAC-3'<br>A: 5'-GACACCCGTCAGACCAGGAA-3'
**Mouse p22phox**	NM_007806.3	S: 5'-TGGCCTGATTCTCATCACTGG-3'<br>A: 5'-GGGACAACTCCACAGAAACTC-3'
**Mouse MCP-1**	NM_011333.3	S: 5'-ACTGAAGCCAGCTCTCTCTTCCTC-3'<br>A: 5'-TTCCTTCTTGGGGTCAGCACAGAC-3'
**Mouse TNFα**	NM_013693.3	S: 5'-GGTGCCTATGTCTCAGCCTCT-3'<br>A: 5'-CATCGGCTGGCACCACTAGTT-3'
**Mouse NOS2**	NM_010927.4	S: 5'-CAGAGGACCCAGAGACAAGC-3'<br>A: 5'-TGCTGAAACATTTCCTGTGC-3'
**Mouse β-Actin**	NM_007393.5	S: 5'-CGTGAAAAGATGACCCAGATCA-3'<br>A: 5'-TGGTACGACCAGAGGCATACAG-3'
**Human Nox1**	NM_013955	S: 5'-CACAAGAAAAATCCTTGGGTCAA-3'<br>A: 5'-GACAGCAGATTGCGACACACA-3'
**Human Nox2**	KU178009.1	S: 5'-TCACTTCCTCCACCAAAACC-3'<br>A: 5'-CACCTTCTGTTGAGATCGCC-3'
**Human Nox4**	NM_016931	S: 5'-TGGCTGCCCATCTGGTGAATG-3'<br>A: 5'-CAGCAGCCCTCCTGAAACATGC-3'
**Human Nox5**	NM_024505	S: 5'-CAGGCACCAGAAAAGAAAGCAT-3'<br>A: 5'-ATGTTGTCTTGGACACCTTCGA-3'
**Human p22phox**	NM_000101.4	S: 5'-GTTTGTGTGCCTGCTGGAGT-3'<br>A: 5'-TGGGCGGCTGCTTGATGGT-3'
**Human β-Actin**	NM_001101	S: 5'-CTGGCACCCAGCACAATG-3'<br>A: 5'- GCCGATCCACACGGAGTACT-3'

Table S2. Sequences of sense (S) and antisense (A) oligonucleotide primers used in real-time PCR assays.

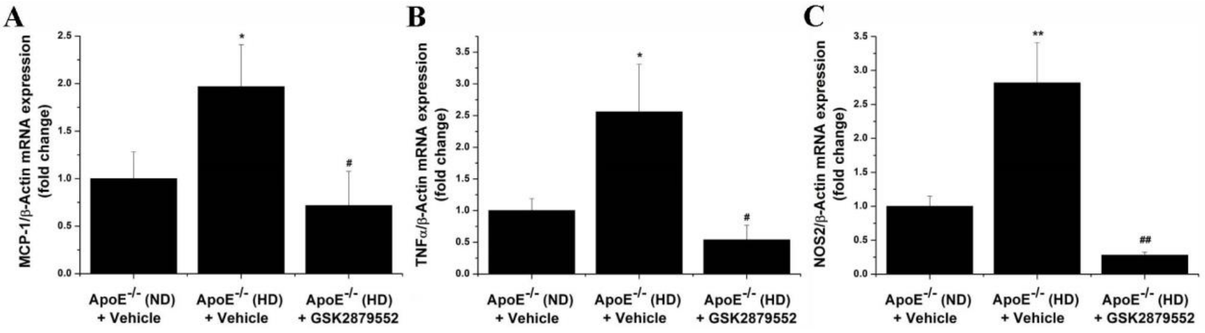

Figure S1. LSD1 blockade suppresses the up-regulation of (A) MCP-1, (B) TNFα, and (C) NOS2 transcript levels in the atherosclerotic aorta of ApoE-/- (HD) mice. n=3, *P < 0.05, **P < 0.01. P-values were taken in relation to vehicle-treated ApoE-/- (ND) condition. # P < 0.05, ## P < 0.01. P-values were taken in relation to vehicle-treated ApoE-/- (HD) condition.

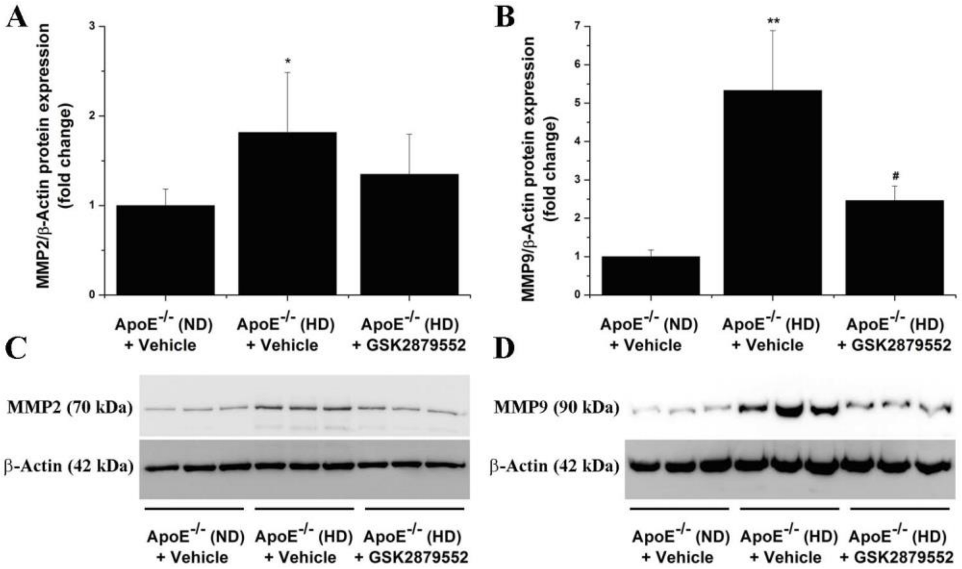

Figure S2. (A, B) LSD1 mediates the up-regulation of MMP9, but not MMP2 protein levels in the atherosclerotic aorta of ApoE-/- mice. (C, D) Representative immunoblots depicting the changes in MMP2 and MMP9 protein expression levels function of experimental condition. n=3-6, *P < 0.05, **P < 0.01. P-values were taken in relation to vehicle-treated ApoE-/- (ND) condition. # P < 0.05. P-value was taken in relation to vehicle-treated ApoE-/- (HD) condition.

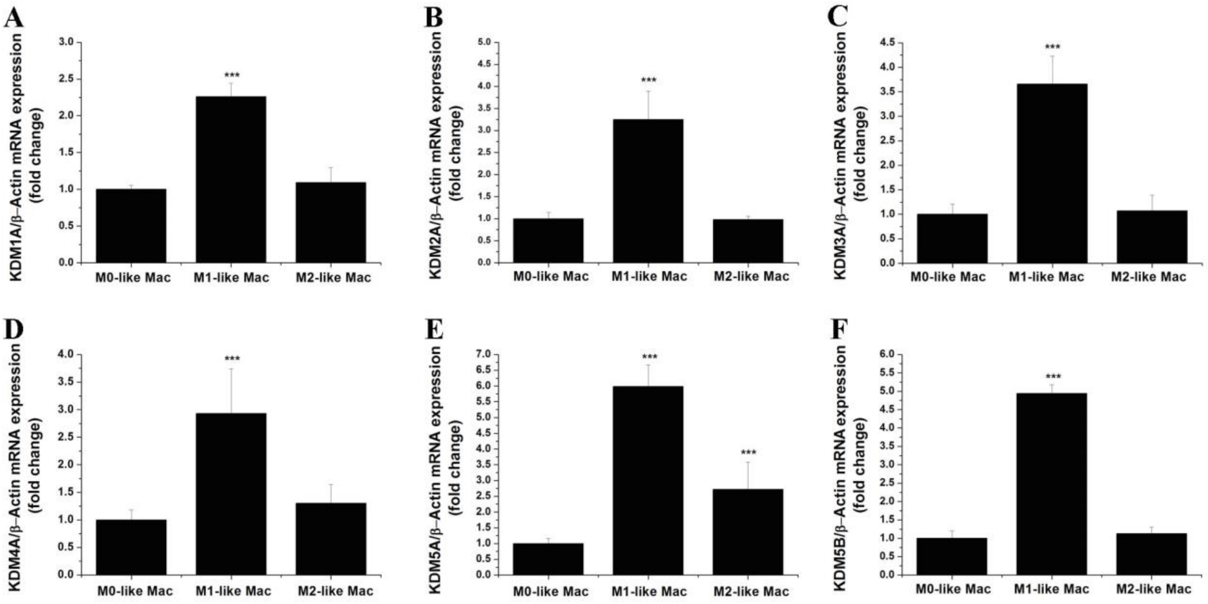

Figure S3. Elevated mRNA expression levels of (A) LSD1/KDM1A, (B) KDM2A, (C) KDM3A, (D) KDM4A, (E) KDM5A, and (F) KDM5B subtypes are associated with a pro-inflammatory macrophage phenotype in vitro. n=4, ***$P < 0.001$. P-values were taken in relation to resting macrophage phenotype (M0-like Mac) condition.

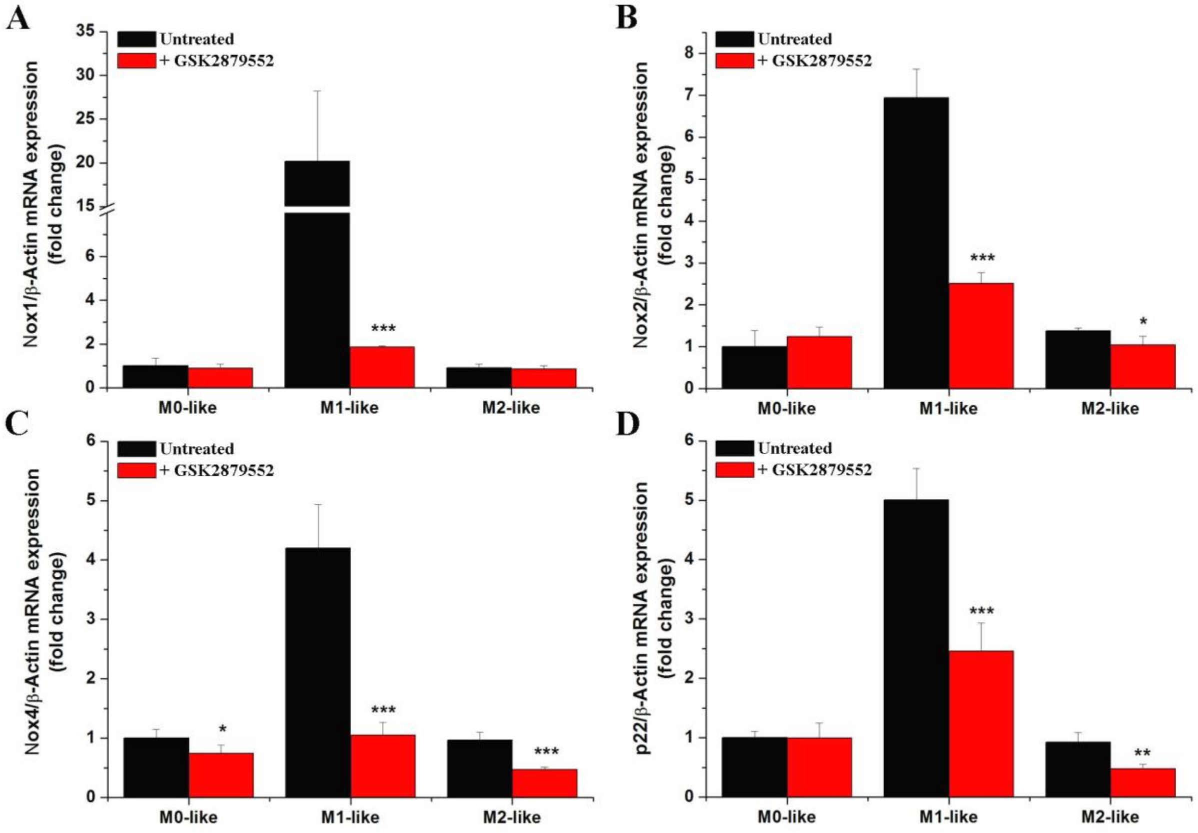

Figure S4. GSK2879552-induced pharmacological inhibition of LSD1 function suppresses the up-regulation of (A) Nox1, (B) Nox2, (C) Nox4, and (D) p22phox mRNA levels in cultured pro-inflammatory macrophages (M1-like Mac). n=4, *P < 0.05, **P < 0.01, ***P < 0.001. P-values were taken in relation to untreated conditions.

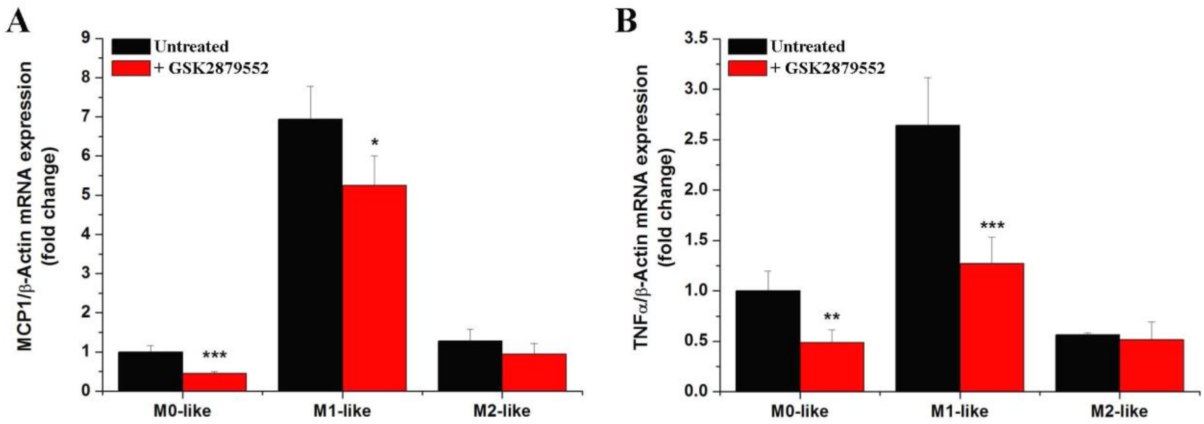

Figure S5. LSD1 blockade reduces the up-regulation of (A) MCP-1 and (B) TNFα transcript levels in cultured pro-inflammatory macrophages (M1-like Mac). n=4, *P < 0.05, **P < 0.01, ***P < 0.001. P-values were taken in relation to untreated conditions.
